# Supplementary material for: Human neuronal networks on micro-electrode arrays are a highly robust tool to study disease-specific genotype-phenotype correlations in vitro
Source: Stem Cell Reports. 2021 Jul 29;16(9):2182–96. doi: 10.1016/j.stemcr.2021.07.001 (PMC8452490; doi:10.1016/j.stemcr.2021.07.001)
Supplement: Document S1. Supplemental experimental procedures, Figures S1–S4, and Tables S1–S3 [file mmc1.pdf]

Stem Cell Reports, Volume *16*

Supplemental Information

Human neuronal networks on micro-electrode arrays are a highly robust tool to study disease-specific genotype-phenotype correlations *in vitro*

Britt Mossink, Anouk H.A. Verboven, Eline J.H. van Hugte, Teun M. Klein Gunnewiek, Giulia Parodi, Katrin Linda, Chantal Schoenmaker, Tjitske Kleefstra, Tamas Kozicz, Hans van Bokhoven, Dirk Schubert, Nael Nadif Kasri, and Monica Frega

Supplemental material

Supplemental items

Figure S1 - Variability in neuronal network phenotypes between control lines.
Figure S2 - Average burst shapes of control lines.
Figure S3 - Connectivity in control neuronal networks.
Figure S4 - Additional parameters affected in MELAS and KS.

Supplemental tables

Table S1 – Explanation of extracted MEA parameters.
Table S2 – Stability of MEA parameters.
Table S3 – Power calculations on MEA data.
Table S4 – Statistics per figure (added as separate excel file)

Supplemental experimental procedures

hiPSC line origin and generation
MEA recordings and data analysis
Principal component analysis

Supplemental figures

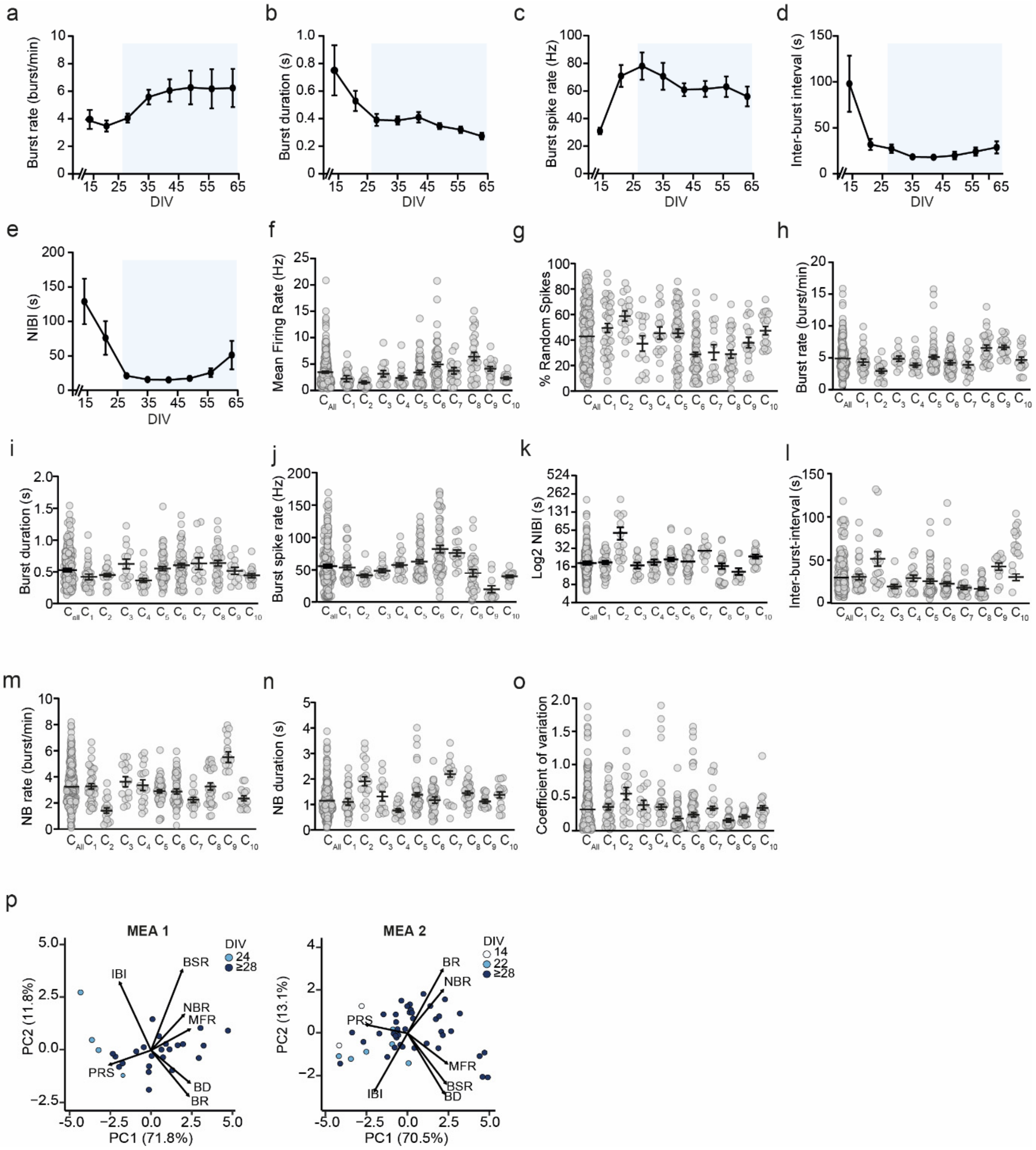

Figure S1. Variability in neuronal network phenotypes between control lines, related to Figure 1. (**a-e**) Neuronal network parameters (representative line C6) develop to reach a certain plateau after DIV 27 (blue box) for (**a**) BR, (**b**) BD, (**c**) BSR, (**d**) IBI and (**e**) NIBI. (**f-o**) Comparison of the MEA parameters (**f**) MFR, (**g**) PRS, (**h**) BR, (**i**) BD, (**j**) BSR, (**k**) NIBI (on log2 scale), (**l**) IBI, (**m**) NBR, (**n**) NBD, (**o**) CV_{NIBI} between all 10 control lines. Kruskal Wallis Anova with Dunn's correction for multiple testing was used to compare between control lines (**Table S4**). (**p**) PCA plots on all parameters

showing data of one control line (C_6) pooled and color-coded by DIV (two independent MEA plates are analyzed). DIV = days in vitro, BR = mean burst rate, BD = mean burst duration, BSR = burst spike rate, IBI = inter-burst interval, NIBI = Network burst IBI, MFR = mean firing rate, PRS = percentage of random spike, NBR = network burst rate, NBD = network burst duration, CV_{NIBI} = coefficient of variation on NIBI.

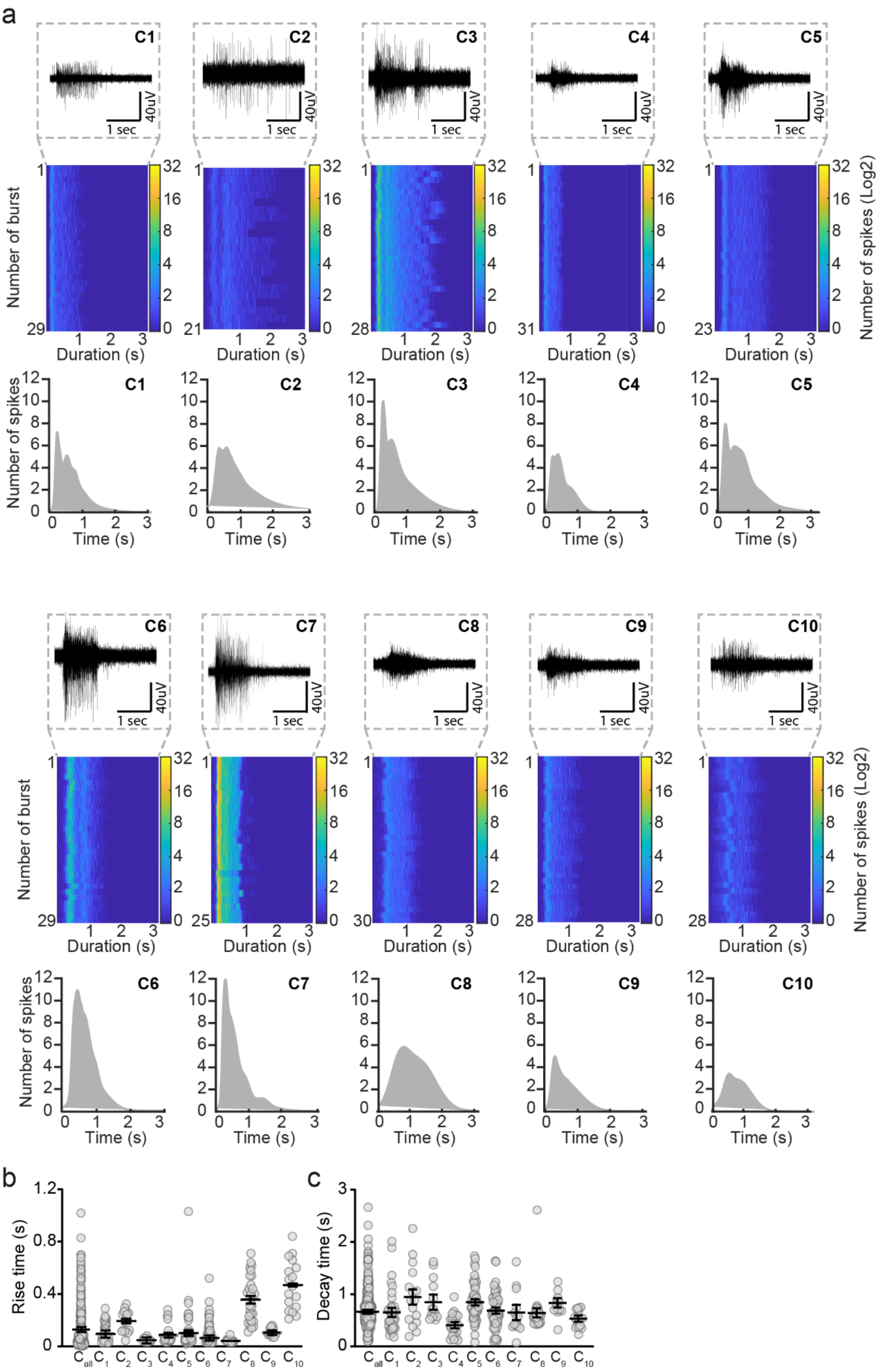
a
C1
C2
C3
C4
C5
C6
C7
C8
C9
C10
40uV
1 sec
Number of burst
Duration (s)
Number of spikes (Log2)
Number of spikes
Time (s)
b
Rise time (s)
c
Decay time (s)
C_{all} C_1 C_2 C_3 C_4 C_5 C_6 C_7 C_8 C_9 C_{10}

Figure S2. Average burst shapes of control lines, related to Figure 1. (**a**) Representative network burst trace (zoom in) and alignment from one recording of control lines. Bottom panel: average burst shape from all recordings (Sample size n for C_1 = 38, C_2 = 15, C_3 = 14, C_4 = 23, C_5 = 55, C_6 = 58, C_7 = 14, C_8 = 30, C_9 = 12 and C_{10} = 17). Average burst shapes were used to calculate the (**b**) RT and (**c**) DT. Kruskal Wallis ANOVA with Dunn's correction for multiple testing was used to compare between control lines (**Table S4**). RT = Rise time, DT = decay time.

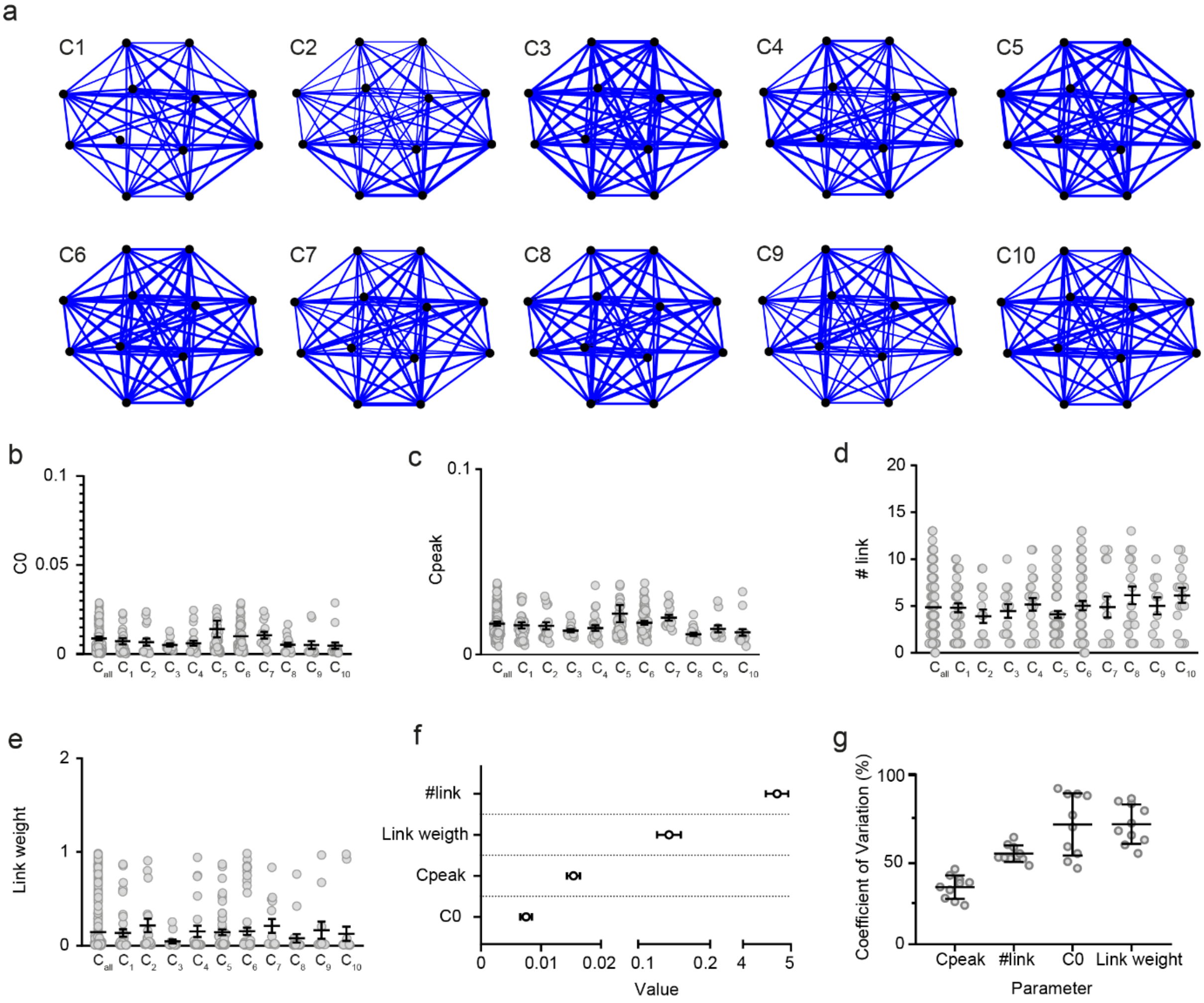

Figure S3. **Connectivity in control neuronal networks, related to Figure 1.** (**a**) Representative connectivity maps showing the links (blue lines) and their strengths (magnitude of the blue lines) (**b-e**) Comparison of the MEA parameters (**b**) C_0, (**c**) C_{peak}, (**d**) number of links and (**e**) weight of links between all 10 control lines. (**f**) Graph showing the range in which MEA parameters C_0, C_{peak}, link weight and number of links of all 10 control lines behave (mean ± 95% confidence interval). Values are first averaged per control line, and then averaged across all control lines. (**g**) Percent coefficient of variation explaining the stability of the respective MEA parameter across all 10 control lines (mean ± standard deviation of the mean). N = 278 wells. Kruskal Wallis ANOVA with Dunn's correction for multiple testing was used to compare between control lines (**Table S4**).

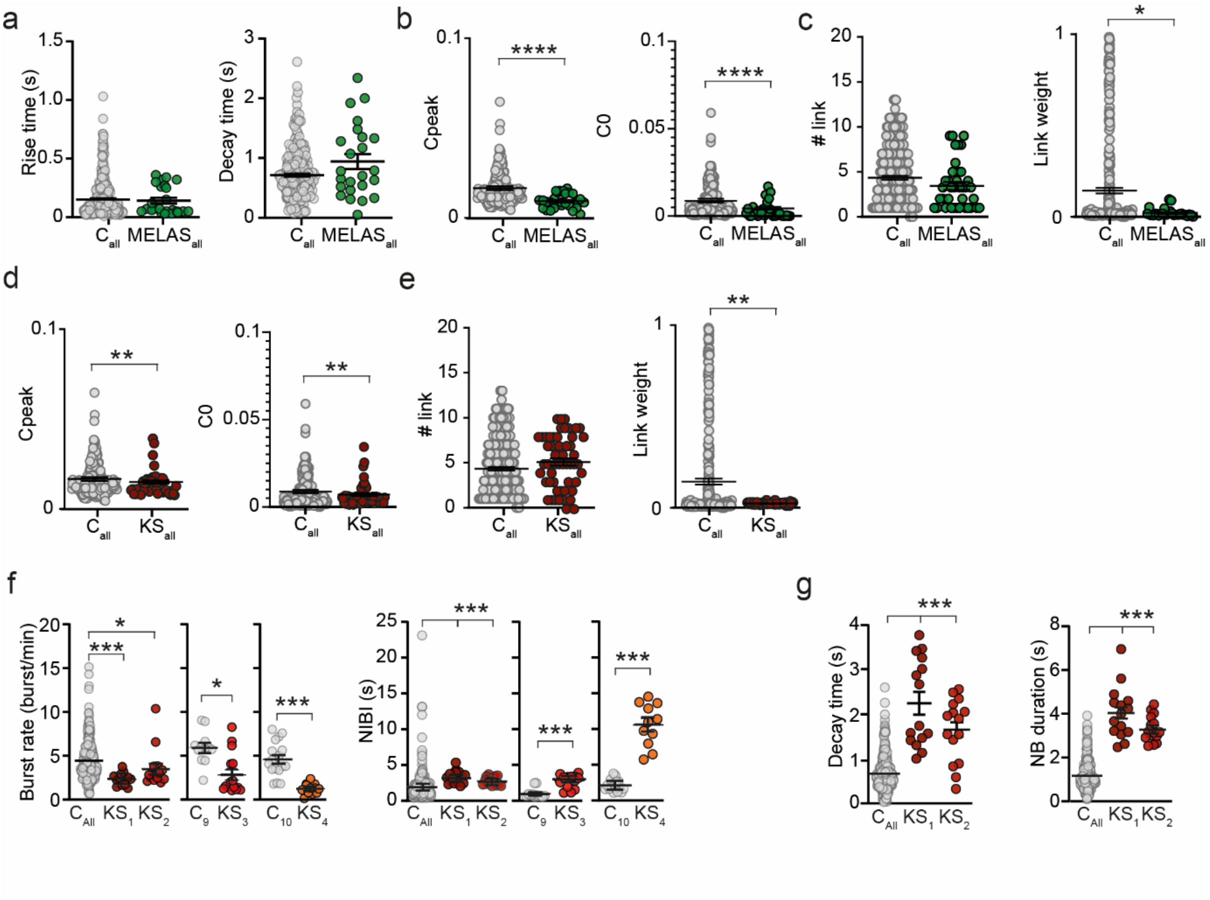

Figure S4. **Additional parameters affected in MELAS and KS, related to Figure 3.** (**a-c**) Comparison of MEA parameters (**a**) RT and DT, (**b**) C_{peak}, C_0 and (**c**) link number and link weight for MELAS samples versus all controls (Mann Whitney U test with Bonferroni correction for multiple testing was used to compare between patient lines and controls). (**d-e**) Comparison of MEA parameters (**d**) C_{peak}, C_0 and (**e**) link number and link weight for KS samples versus all controls (Mann Whitney U test with Bonferroni correction for multiple testing was used to compare between patient lines and controls). (**f-g**) Comparison of MEA parameters (**f**) BR and NIBI for all KS_1 and KS_2 compared to all controls, and for KS_3 and KS_4 compared to their isogenic controls and (**g**) DT and NBD for KS_1 and KS_2 compared to all controls (Kruskal Wallis Anova with Dunn's correction for multiple testing was used to compare between control lines). (**Table S4**). $p = 0.05$ *, $p = 0.01$ **, $p = 0.001$ *** and $p<0.0001$ ****. DIV = days in vitro, BR = mean burst rate, NIBI = Network burst inter-burst-interval, RT = Rise time, DT = decay time, BD = burst duration, NBR = network burst rate, NBD = network burst duration. All means, *p*-values and used statistic tests are reported in Table S4.

Supplemental tables

Parameter	*Short name*	*Unit*	*Explanation*
Mean firing rate	MFR	Spikes/s	The MFR is a measure of all spikes detected in each electrode during time, which is averaged for all 12 electrodes in a well.
Percentage of random spikes	PRS	%	The PRS represents the percentage of all spikes that are not organized into a random burst, or a network burst.
Mean burst rate	MBR	Burst/ minute	A burst is detected when at least 4 spikes are 50 ms or less spaced from each other. The MBR is a measure of all burst detected in each channel in time, which is averaged for all 12 electrodes in a well.
Mean burst duration	MBD	s	The MBD represents the duration of the burst.
Inter-burst interval	IBI	s	The IBI the interval between two consecutive bursts.
Burst spike rate	BSR	Spikes/s	The BSR is the number of spikes detected in a burst divided by the duration of the burst.
Network burst rate	NBR	Network burst/ minute	When a burst is occurring in more than 6 channels at the same time, and 6 are time locked, these bursts are classified as network burst.
Network burst duration	NBD	s	The NBD represents the duration of the network burst.
Network inter-burst interval	NIBI	s	The NIBI is the interval between two consecutive network bursts.
Coefficient of variation on NIBI	CV_{NIBI}		The coefficient of variation is calculated by dividing the standard deviation of all NIBIs to the mean. The value ranges between 0 (very regular network burst) to 1 (very irregular network burst).
Average burst shape			The average burst shape is an averaged histogram representing the number of spikes occurring into all network burst detected in one well.
Rise time	RT	s	The rise time is extracted by the average burst shape histogram by fitting it with a Gaussian and calculating the slope between the 20 and 80% of the peak on the rising edge.
Decay time	DT	s	The decay time is extracted by the average burst shape histogram by fitting it with a Gaussian and calculating the slope between 80 and 20% of the peak on the falling edge.
C_0			C_0 represent the value of the cross correlogram function in the central time bin and indicates the degree of synchronization between two electrodes.
C_{peak}			C_{peak} represent the peak value of the cross correlogram and indicates the degree of correlation between two electrodes.
Number of links			The number of links indicates the total number of connections between all MEAs electrodes.
Link weight			The link weight indicates the averaged strength of all links detected between all MEAs electrodes

Supplemental table 1: **Explanation of extracted MEA parameters.**

Panel		*MFR*	*PRS*	*MBR*	*MBD*	*BSR*	*IBI*	*NBR*	*NBD*	*NIBI*	*CV_{NIBI}*	*RT*	*DT*
i	Mean	3.5	41.3	4.8	0.51	58.5	27.47	3.2	1.28	22.68	0.3	0.15	0.71
	SEM	0.2	1.3	0.1	0.02	1.8	1.20	0.1	0.04	1.16	0.0	0.01	0.02
	Min	0.4	3.0	1.0	0.12	8.5	5.08	0.3	0.19	7.85	0.0	0.01	0.07
	Max	20.9	92.3	15.5	1.53	170.2	131.99	7.0	4.02	222.80	1.9	1.03	2.61
j	Mean	62.96	46.37	39.38	42.88	39.63	58.21	38.37	40.60	43.79	74.77	58.49	48.57
	STD	17.39	14.53	9.337	7.749	20.14	13.34	9.218	10.56	9.092	18.73	26.93	12.46
	Min	29.38	26.40	26.37	28.63	17.73	33.71	28.25	20.10	30.54	47.21	28.61	29.27
	Max	87.68	73.74	57.78	51.33	84.03	80.75	61.30	54.24	93.80	103.7	141.3	64.41

		C_{peak}	*C_0*	*# link*	*Link weight*
f	Mean	0.007	0.004	4.712	0.143
	SEM	0.001	0.0009	0.234	0.016
	Min	0.010	0.004	3.647	0.045
	Max	0.022	0.014	5.889	0.216
g	Mean	46.79	75.3	60.86	76.1
	STD	13.75	26.57	9.736	13.13
	Min	25.45	48.76	56.98	63.7
	Max	67.95	92.5	75.05	85.1

Supplemental table 2: Stability of MEA parameters, related to Figure 1i-j, 3f-g: Values of MEA parameters presented **in Figure 1i, j** and **Figure S3f, g. Panel i, f:** Range of each MEA parameters in which controls behave**. Panel j, g:** Percentage of variation of each MEA parameter**.**

		Post hoc power calculation		*A priori power calculation*	
Compared lines	**Top parameters**	**# wells used for calculation**	**Power**	**# wells needed for power 0.9**	**Effect size**
C9 vs KS3	BR	C9 = 12	0.999	12	1.9
	BD	KS3 =16	0.999	12	1.9
	NBR		1	6	3
	NBD		0.999	8	2.7
	NIBI		0.999	10	2.3
C10 vs KS4	MFR	C10 = 17	0.999	12	2
	BR	KS4 =12	1	6	3
	NBR		0.98	14	2
	NBD		0.97	8	2.8
	NIBI		1	6	3.4
C4 versus M2	MFR	C4 = 23	0.999	12	2
	PRS	M2 = 23	1	10	2.1
	BD		0.998	20	1.4
	BSR		0.999	16	1.5
	NBR		1	6	3
C5 versus M3	MFR	C5 = 55	1	12	1.9
	PRS	M3 = 68	1	12	1.9
	BD		0.999	24	1.4
	BRS		1	22	1.3
	NBR		1	12	2
C2 versus M1	PRS	C2 = 17	1	8	2.8
	BD	M1 = 24	0.999	16	1.6
	NBR		0.927	6	1
	BSR		0.997	22	1.5
All controls vs KS	BR	C_{all} = 278	1	14	2
	BD	KS_{all} = 58	1	12	2.1
	NBR		1	14	2
	NBD		1	14	1.9
	NIBI		0.999	10	2.55
	DT		0.999	12	2.27
All controls vs MELAS	MFR	C_{all} = 278	1	12	1.9
	PRS	$MELAS_{all}$ = 115	1	8	2.5
	BD		1	8	2.78
	BSR		1	10	2.3
	NBR		1	6	3

Supplemental table 3: Power calculations on MEA data. Left: post hoc power calculation on PCA parameters that describe the patient phenotype. Alpha is 0.05. Right: a priori Power calculation to calculate the sample size needed to achieve a power of 0.9 using the PCA parameters that describe the patient phenotype. Effect size represents the magnitude of the difference between populations.

Supplemental table 4 (added as separate excel file): Statistics per figure. Sample size n represents the number of recorded MEA wells between DIV27-35. All data represent means ± SEM. Statistical tests used per figure are reported in the figure legends.

Supplemental experimental procedures

hiPSC line origin and generation

C_1 originated from a 36-year old female (Kondo et al., 2017; Okita et al., 2011), reprogrammed using episomal vector-based reprogramming of the Yamanaka transcription factors *Oct4*, *c-Myc*, *Sox2* and *Klf4* (Takahashi & Yamanaka, 2006), showing no karyotypical malformations. Control line **C_6** was derived from a 30-year old male (Mandegar et al., 2016; Miyaoka et al., 2014) and reprogrammed using episomal vector-based reprogramming of the Yamanaka factors and was tested for genetic integrity using SNP assay (Frega et al., 2019). Control line **C_7** was derived from a 41-year-old-female and reprogrammed using the Simplicon™ reprogramming kit (Millipore). Overexpression of the Yamanaka factors was introduced by a non-integrative, non-viral one-step transfection. Genomic stability was checked using STR analysis. Control line **C_8** was derived from a 9-year old male and reprogrammed using retroviral-vector based reprogramming of the Yamanaka factors and tested for pluripotency and genomic integrity based on SNP arrays.

The KS isogenic patient-control hiPSC set consisting of control line **C_9 and KS_3** was derived from a 34-year old female with a mosaic heterozygous 233 kbp deletion of chromosome 9, including the *EHMT1* and *CACNA1B* gene, diagnosed with KS (Frega et al., 2019). Both clones were reprogrammed using retro-viral vectors expressing the Yamanaka factors and tested for genomic integrity based on SNP array (Frega et al., 2019). The second KS isogenic patient-control hiPSC set, consisting of control line **C_{10} and KS_4** was previously derived from a healthy 51-year old male and reprogrammed using expression of Yamanaka factors by non-integrating Sendai virus. KS_4 was generated using CRISPR/Cas 9 technology to induce a heterozygous *EHMT1* mutation to mimic KS, as described previously, producing an isogenic set. Both lines were tested for genomic integrity based on SNP array (Frega et al., 2019). In addition, we included two KS patient hiPSC lines, **KS_1 and KS_2,** which were previously characterized and derived from a 13-year old and a 12-year-old female, respectively, diagnosed with KS. HiPSC clones were obtained by reprogramming of retroviral expression of the Yamanaka factors (Frega et al., 2019).

The MELAS syndrome isogenic patient-control hiPSC sets consisting of control line **C_2, C_3, C_4 and C_5 and patient lines M_1 and M_2** were generous gifts form Esther-Perales Clemente and Timothy Nelson. **C_2 and M_1** were derived from a 17-year-old female, and **C_4 and M_2** form a 45-year-old female. Both donors were diagnosed with MELAS syndrome, harboring the pathogenic variant m.3243A>G. Reprogramming of fibroblasts to hiPSCs resulted in clones with varying levels of m.3243A>G heteroplasmy (Klein Gunnewiek et al., 2020; Perales-Clemente et al., 2016). Control lines C_2 and C_4 originated from hiPSC clones with confirmed 0% heteroplasmy, while M_1 and M_2 had 66% of m.3243A>G heteroplasmy and 80% heteroplasmy, respectively. Control line **C_3** was derived from a 30-

year-old male with MELAS syndrome and was confirmed to have 0% heteroplasmy upon reprogramming to hiPSCs. All lines were reprogrammed using CytoTune-iPS Sendai Reprogramming Kits according to manufacturer's instructions (Invitrogen, A13780-02, A16517, A16518) and were previously characterized and had a normal karyotype (Perales-Clemente et al., 2016). MELAS syndrome isogenic patient-control hiPSC set, consisting of control line **C_5 and M_3** was derived from a 42-year-old male with MELAS syndrome with a pathogenic variant m.3243A>G. Reprogramming through lentiviral transduction of the Yamanaka transcription factors resulted in hiPSC clones with varying levels of m.3243A>G heteroplasmy. C_5 originated from an hiPSC clone with confirmed 0% of m.3243A>G heteroplasmy and M_3 originated from an hiPSC clone with 70% heteroplasmy. Both C_5 and M_3 were previously characterized and had normal karyotypes (Klein Gunnewiek et al., 2020).

MEA recordings and data analysis

Data analysis using Multiwell-Analyzer. The mean firing rate (MFR) (Hz) was calculated for each well individually by averaging the firing rate of each separate channel by all the active channels of the well. Bursts were detected using the Multiwell analyzer build-in burst detection algorithm. The algorithm was set to define bursts if 4 spikes were in close proximity with a maximum of 50 ms inter spike interval (ISI) to start a burst, and a maximum of 50 ms ISI to end a burst, with a minimum of 100 ms inter burst interval (IBI). Network bursts were defined when at least 50% of all channels simultaneously displayed a burst. In rare cases, we observed synchronous events which were not properly identified as network bursts **(Fig. 2g)**. It was however clear that the observed pattern of the synchronous events was similar to control networks in which network bursts were properly detected. When the network burst detection was insufficient the network bursts detection was decreased down to, but not further than at least 25% of all channels participating in the network burst. The percentage of random spikes (PRS) was defined by calculating the percentage of spikes that neither belonged to a burst nor a network burst. The IBI, and network burst IBI (NIBI) were calculated by the subtraction of the time stamp of the beginning of each burst or network burst, respectively, from the time stamp of the ending of the previous. To calculate the IBI, the mean IBI per channel was calculated and averaged across all channels. The NIBI is calculated by averaging the found NIBIs in one well. To explain the regularity at which a network burst occurs, the coefficient of variation (CV) was calculated by calculating the standard deviation of all NIBIs and dividing it by the mean of all NIBIs.

Analysis of the average burst shape. The generation of average burst shapes for each control was performed using adapting scripts and functions implemented in MATLAB (Melé and Rinn, 2016; Van De Vijver et al., 2019). Spike trains containing all events detected in the 12 electrodes were binned. Network burst were detected from a spike train containing the events present in all channels

by using an inter-spike interval threshold (30 ms). All network bursts that have a duration of less than 100 ms were removed from the detection. For each well, the average burst shape was calculated by aligning all the network bursts detected to the longest network burst and by summing all spikes (bin: 10 ms). Then, we calculated the average burst shape for each line by averaging all the histograms of the individual wells. We fitted the curves with Gaussian distribution to obtain smoother profiles using the build in fit function. The rise and decay times were calculated as the absolute difference between the time at 20% and 80% before and after the peak, respectively. Slopes of rise and decay time were subsequently calculated using the build in polyfit and polyval functions.

Correlation analysis. We obtained connectivity matrix for each culture by applying the Filtered Cross-Correlation algorithm (Pastore, Massobrio, et al., 2018) available in the free software SPICODYN (Pastore, Godjoski, et al., 2018). Cross correlograms were obtained using a bin size of 0.1 ms and a correlation window of 150 ms. Matrices were then analyzed using custom script in MATLAB. We evaluated the C_{peak} (i.e. maximum value of the -cross-correlation function), C_0 (i.e. value of the cross-correlation function in the central bin), total number of links (i.e. the number of functional connections within the nodes of the same network) and the link weight (i.e. the average strength of the identified connections).

Principal component analysis

Principal component analysis (PCA) was performed on data from 12 MEA parameters (MFR, PRS, BR, BD, BSR, IBI, NBR, NBD, NIBI, CV_{NIBI}, RT, and DT, see Table S1) for all control samples (n_{wells} = 278, N_{plates} = 47). Separately, PCA was performed for controls including samples from either KS (n_{wells} = 58, N_{plates} = 9) or MELAS (n_{wells} = 112, N_{plates} = 23) neurons. PCA on KS versus control samples was performed on data from all 12 MEA parameters. PCA was performed for each KS isogenic patient-control set separately as well, using the same MEA parameters. PCA on MELAS versus control samples was performed on data from 7 MEA parameters (MFR, PRS, BR, BD, BSR, IBI, and NBR), since many samples did not show network bursts (i.e. NBD, NIBI, CV_{NIBI}, RT, and DT could not be calculated). PCA was performed for each MELAS isogenic patient-control set separately as well, using the same MEA parameters.

PCA in **Fig. 2i** was performed for samples from line C1 separately to test the effect of astrocyte batch and MEA plate, using data from all 12 MEA parameters. Samples from C1 were measured using three different astrocyte batches and 2-3 different MEA plates per astrocyte batch. PCA was performed on all C1 samples (n_{wells} = 27, N_{plates} = 7, N_{astro} = 3) to check the effect of astrocyte batch and MEA batch. ANOVA was used to evaluate the significance of the linear models (parameter ~ MEA plate and parameter ~ astrocyte batch) to determine the effect of astrocyte batch and MEA batch,

separately, on each MEA parameter. Statistics are reported in **Table S4**. In **Fig. 2j** the percentage of variance explained by astrocyte batch and MEA batch on each parameter is shown for all parameters, calculated by dividing the sum of squares by the total sum of squares (x 100) for each linear model separately.

Furthermore, PCA in **Fig. S1p** was performed for samples from line C6 that were measured up to DIV62, to check whether samples from early DIVs cluster away from samples at a higher DIVs. This was performed for two MEA plates separately, for which data was available between DIV14-56 (n_{wells} = 7) and DIV24-62 (n_{wells} = 4), using data from 7 MEA parameters (MFR, PRS, BR, BD, BSR, IBI, and NBR), since at an early time point (almost) no network bursts are detected (i.e. NBD, NIBI, CV_{NIBI}, RT, and DT could not be calculated).

References

Frega, M., Linda, K., Keller, J. M., Gümüş-Akay, G., Mossink, B., van Rhijn, J. R., Negwer, M., Klein Gunnewiek, T., Foreman, K., Kompier, N., Schoenmaker, C., van den Akker, W., van der Werf, I., Oudakker, A., Zhou, H., Kleefstra, T., Schubert, D., van Bokhoven, H., & Nadif Kasri, N. (2019). Neuronal network dysfunction in a model for Kleefstra syndrome mediated by enhanced NMDAR signaling. *Nature Communications*, *10*(1), 1–15. https://doi.org/10.1038/s41467-019-12947-3

Klein Gunnewiek, T. M., Hugte, E. J. H. Van, Frega, M., Guardia, G. S., Foreman, K. B., Panneman, D., Mossink, B., Linda, K., Keller, J. M., Schubert, D., Cassiman, D., Morava, E., Rodenburg, R., Perales-Clemente, E., Nelson, T. J., Kasri, N. N., & Kozicz, T. (2020). Mitochondrial dysfunction impairs human neuronal development and reduces neuronal network activity and synchronicity. *Cell Reports*, 720227. https://doi.org/10.1101/720227

Kondo, T., Imamura, K., Funayama, M., Tsukita, K., Miyake, M., Ohta, A., Woltjen, K., Nakagawa, M., Asada, T., Arai, T., Kawakatsu, S., Izumi, Y., Kaji, R., Iwata, N., & Inoue, H. (2017). iPSC-Based Compound Screening and In Vitro Trials Identify a Synergistic Anti-amyloid β Combination for Alzheimer's Disease. *Cell Reports*, *21*(8), 2304–2312. https://doi.org/10.1016/j.celrep.2017.10.109

Mandegar, M. A., Huebsch, N., Frolov, E. B., Shin, E., Truong, A., Olvera, M. P., Chan, A. H., Miyaoka, Y., Holmes, K., Spencer, C. I., Judge, L. M., Gordon, D. E., Eskildsen, T. V., Villalta, J. E., Horlbeck, M. A., Gilbert, L. A., Krogan, N. J., Sheikh, S. P., Weissman, J. S., … Conklin, B. R. (2016). CRISPR Interference Efficiently Induces Specific and Reversible Gene Silencing in Human iPSCs. *Cell Stem Cell*, *18*(4), 541–553. https://doi.org/10.1016/j.stem.2016.01.022

Miyaoka, Y., Chan, A. H., Judge, L. M., Yoo, J., Huang, M., Nguyen, T. D., Lizarraga, P. P., So, P. L., & Conklin, B. R. (2014). Isolation of single-base genome-edited human iPS cells without antibiotic selection. *Nature Methods*, *11*(3), 291–293. https://doi.org/10.1038/nmeth.2840

Okita, K., Matsumura, Y., Sato, Y., Okada, A., Morizane, A., Okamoto, S., Hong, H., Nakagawa, M.,

Tanabe, K., Tezuka, K. I., Shibata, T., Kunisada, T., Takahashi, M., Takahashi, J., Saji, H., & Yamanaka, S. (2011). A more efficient method to generate integration-free human iPS cells. *Nature Methods*, *8*(5), 409–412. https://doi.org/10.1038/nmeth.1591

Pastore, V. P., Godjoski, A., Martinoia, S., & Massobrio, P. (2018). SpiCoDyn: A Toolbox for the Analysis of Neuronal Network Dynamics and Connectivity from Multi-Site Spike Signal Recordings. *Neuroinformatics*, *16*(1), 15–30. https://doi.org/10.1007/s12021-017-9343-z

Pastore, V. P., Massobrio, P., Godjoski, A., & Martinoia, S. (2018). *Identification of excitatory-inhibitory links and network topology in large-scale neuronal assemblies from multi-electrode recordings*. https://doi.org/10.1371/journal.pcbi.1006381

Perales-Clemente, E., Cook, A. N., Evans, J. M., Roellinger, S., Secreto, F., Emmanuele, V., Oglesbee, D., Mootha, V. K., Hirano, M., Schon, E. A., Terzic, A., & Nelson, T. J. (2016). Natural underlying mt DNA heteroplasmy as a potential source of intra-person hi PSC variability . *The EMBO Journal*, *35*(18), 1979–1990. https://doi.org/10.15252/embj.201694892

Takahashi, K., & Yamanaka, S. (2006). Induction of Pluripotent Stem Cells from Mouse Embryonic and Adult Fibroblast Cultures by Defined Factors. *Cell*, *126*(4), 663–676. https://doi.org/10.1016/j.cell.2006.07.024
