# Supplementary material for: Human neuronal networks on micro-electrode arrays are a highly robust tool to study disease-specific genotype-phenotype correlations in vitro
Source: Stem Cell Reports. 2021 Jul 29;16(9):2182–96. doi: 10.1016/j.stemcr.2021.07.001 (PMC8452490; doi:10.1016/j.stemcr.2021.07.001)
Supplement: Document S2. Article plus supplemental information [file mmc3.pdf]

Stem Cell Reports
Article
ISSCR
OPEN ACCESS

Human neuronal networks on micro-electrode arrays are a highly robust tool to study disease-specific genotype-phenotype correlations *in vitro*

Britt Mossink,[1,8,9] Anouk H.A. Verboven,[1,2,9] Eline J.H. van Hugte,[1,3,9] Teun M. Klein Gunnewiek,[1,4] Giulia Parodi,[1] Katrin Linda,[1] Chantal Schoenmaker,[1] Tjitske Kleefstra,[1] Tamas Kozicz,[4,5,6] Hans van Bokhoven,[1,7] Dirk Schubert,[1,7] Nael Nadif Kasri,[1,7,10] and Monica Frega[1,6,10,*]
[1]Department of Human Genetics, Radboudumc, Donders Institute for Brain, Cognition, and Behavior, 6500 HB Nijmegen, the Netherlands
[2]Centre for Molecular and Biomolecular Informatics, Radboudumc, Radboud Institute for Molecular Life Sciences, 6500 HB Nijmegen, the Netherlands
[3]ACE Kempenhaeghe, Department of Epileptology, 5591 VE Heeze, the Netherlands
[4]Department of Medical Imaging, Radboud University Medical Center, 6525 GA Nijmegen, the Netherlands
[5]Department of Laboratory Medicine and Pathology, Mayo Clinic, Rochester, MN 55905, USA
[6]Department of Clinical Genomics, Mayo Clinic, Rochester, MN 55905, USA
[7]Department of Cognitive Neuroscience, Radboudumc, Donders Institute for Brain, Cognition and Behavior, 6500 HB Nijmegen, the Netherlands
[8]Department of Clinical Neurophysiology, University of Twente, 7522 NB Enschede, the Netherlands
[9]These authors contributed equally
[10]Co-senior author
*Correspondence: m.frega@utwente.nl
https://doi.org/10.1016/j.stemcr.2021.07.001

SUMMARY

Micro-electrode arrays (MEAs) are increasingly used to characterize neuronal network activity of human induced pluripotent stem cell (hiPSC)-derived neurons. Despite their gain in popularity, MEA recordings from hiPSC-derived neuronal networks are not always used to their full potential in respect to experimental design, execution, and data analysis. Therefore, we benchmarked the robustness of MEA-derived neuronal activity patterns from ten healthy individual control lines, and uncover comparable network phenotypes. To achieve standardization, we provide recommendations on experimental design and analysis. With such standardization, MEAs can be used as a reliable platform to distinguish (disease-specific) network phenotypes. In conclusion, we show that MEAs are a powerful and robust tool to uncover functional neuronal network phenotypes from hiPSC-derived neuronal networks, and provide an important resource to advance the hiPSC field toward the use of MEAs for disease phenotyping and drug discovery.

INTRODUCTION

In vitro neuronal models have become an important tool to study the complex communication of healthy and diseased neuronal circuits. In particular, the possibility to measure and manipulate the electrical activity exhibited by neuronal populations gives insight into neuronal network development and organization (Kamioka et al., 1996; Maeda et al., 2016; Novellino et al., 2011). Micro-electrode arrays (MEAs) are cell culture dishes with embedded micro-electrodes that allow non-invasive measurement of neuronal network activity. MEAs have been extensively used to measure activity from a range of different neuronal culture systems, for example, primary cell cultures, brain slices, or intact retinas, mainly from rodent origin (McConnell et al., 2012). With the advancements in human induced pluripotent stem cell (hiPSC) technology, the differentiation of human neurons from somatic cells became possible, allowing phenotyping of human neuronal networks. hiPSC-derived neuronal networks on MEA mimic the activity pattern of rodent neuronal networks, including a stable state of synchronized network bursting, suggesting that they successfully develop into functional neuronal networks (Frega et al., 2019; Fukushima et al., 2016; Kayama et al., 2018; Odawara et al, 2014, 2016; Sasaki et al., 2019). In addition, improvements in MEA analysis software simplified the extraction of parameters that describe the pattern of neuronal activity. These advancements in both human neuronal culturing systems and MEA analysis software contributed to the popularity of MEA technology to study neuronal network phenotypes (Deneault et al., 2019; Frega et al., 2019; Klein Gunnewiek et al., 2020; Wainger et al., 2014).

Despite its increasing popularity, MEA technology is not always used to its full potential to investigate hiPSC-derived neuronal network characteristics. hiPSC-derived neuronal networks have not been benchmarked as extensively as rodent neuronal cultures. Because of the lack of standardization, it remains undetermined how changes in cell culture conditions influence batch-to-batch consistency, and whether hiPSC-derived neuronal networks from different lines are comparable (Engle et al., 2018). It is advised to use multiple hiPSC-derived neuronal lines or isogenic sets to reliably determine a disease phenotype, since differences in genetic background between hiPSC donors dominate the variance at the transcriptional level (Germain and Testa, 2017). However, little is known about

© 2021 The Authors.
This is an open access article under the CC BY license (http://creativecommons.org/licenses/by/4.0/).

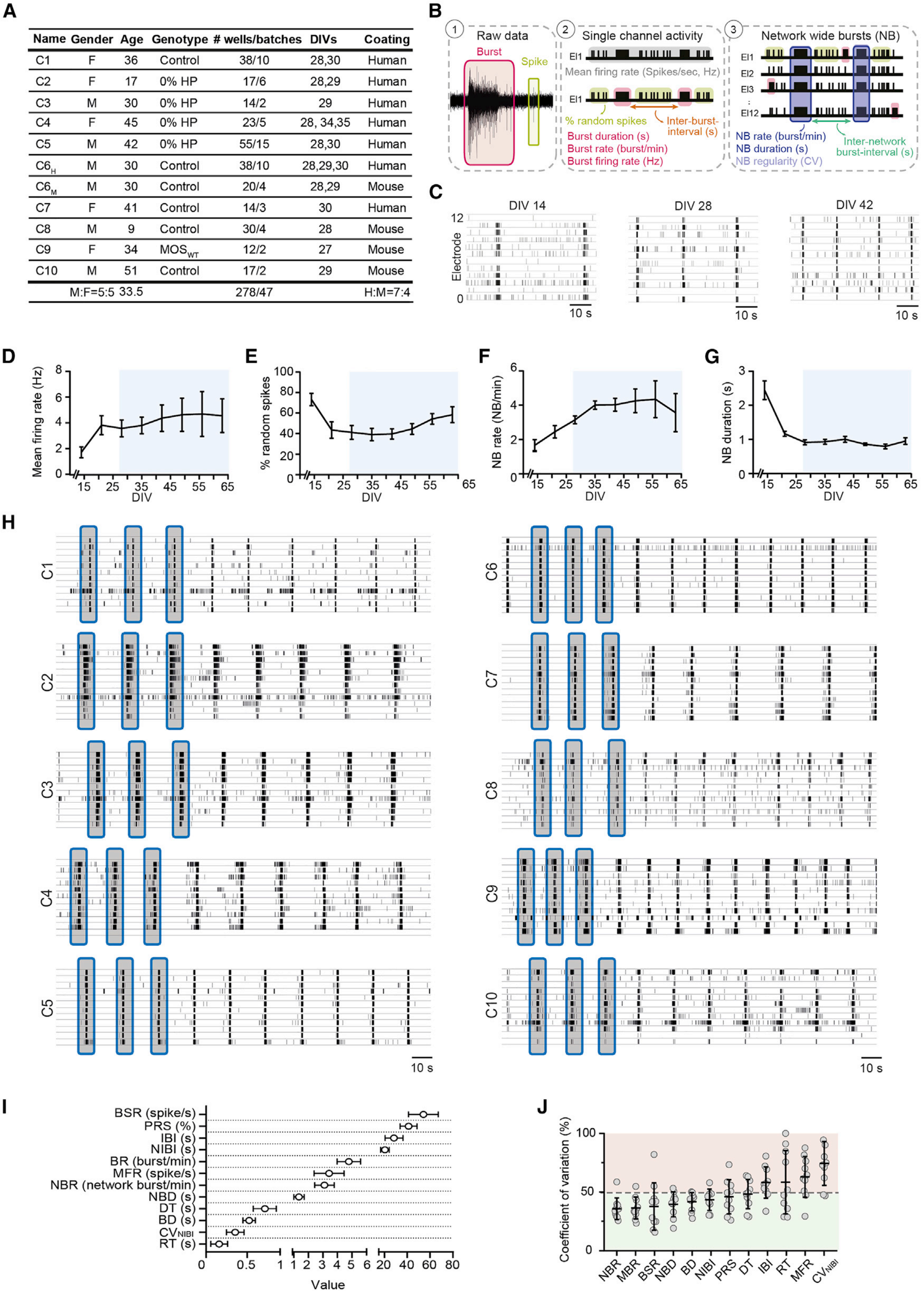

Name	Gender	Age	Genotype	# wells/batches	DIVs	Coating
C1	F	36	Control	38/10	28,30	Human
C2	F	17	0% HP	17/6	28,29	Human
C3	M	30	0% HP	14/2	29	Human
C4	F	45	0% HP	23/5	28, 34,35	Human
C5	M	42	0% HP	55/15	28,30	Human
$C6_H$	M	30	Control	38/10	28,29,30	Human
$C6_M$	M	30	Control	20/4	28,29	Mouse
C7	F	41	Control	14/3	30	Human
C8	M	9	Control	30/4	28	Mouse
C9	F	34	MOS_{WT}	12/2	27	Mouse
C10	M	51	Control	17/2	29	Mouse
	M:F=5:5	33.5		278/47		H:M=7:4

(legend on next page)

the amount of cell lines needed to distinguish a phenotype on MEAs, or about the effect of genetic background on hiPSC-derived neuronal network function. In addition to experimental design, data analysis remains a hurdle, even though the extraction of MEA parameters became easier. Studies often quantify the general neuronal network activity by a single parameter (i.e., mean firing rate), thereby failing to explain the complex network characteristics. Finally, cell culture practices are not always optimized and thus mature networks, showing network synchronicity, are not always obtained. In summary, the question remains how reproducible and comparable MEA recordings are within and between different lines, different researchers, and across different batches or developmental time points, illustrating the need for a quality standard.

Here, we provide a set of recommendations for the design, analysis, and interpretation of hiPSC-derived neuronal networks on MEAs. We performed a meta-analysis of MEA recordings from excitatory neuronal networks generated through one of the most widely used differentiation protocols (i.e., *Ngn2* induction [Frega et al., 2017; Zhang et al., 2013]). Specifically, we used hiPSCs derived from ten healthy subjects (controls), which were cultured by different researchers over a period of several years. We show that different control neuronal networks cultured on MEAs are highly comparable, and identify the most robust MEA parameters to describe neuronal network activity and organization. When pooling data from all control lines, the functional activity of control neuronal networks is not largely influenced by biological differences between donors (i.e., age, sex). Finally, using neuronal networks affected by genetic aberrations causing Kleefstra syndrome (KS) or mitochondrial encephalopathy, lactic acidosis, and stroke-like episodes (MELAS), we show that the MEA platform is a powerful tool to identify genotype-phenotype correlations.

RESULTS

Excitatory neurons derived from healthy subjects show a comparable phenotype on MEA

To investigate if neuronal network activity from hiPSC-derived *Ngn2*-induced excitatory neurons was reproducible, we performed a meta-analysis on MEA data derived from multiple control lines used in our lab (Frega et al., 2019; Klein Gunnewiek et al., 2020; Mossink et al., 2021). The control lines were derived from fibroblast skin biopsies from ten healthy individuals, five males and five females, with a mean age of 33.5 years (Figure 1A), and we extracted 17 parameters in total to describe the neuronal network activity and connectivity (Table S1).

During the first 2 weeks of differentiation, neuronal network activity primarily consisted of random spikes (isolated asynchronous spikes) and bursts (high frequency action potentials), which, during development, organized into network bursts (rhythmic, synchronous events) (Figures 1B and 1C). During maturation, *Ngn2*-induced neuronal networks displayed an increase in firing rate (MFR) and (network) bursting rate ([N]BR), and a decrease in (network) burst duration ([N]BD), and percentage of random spikes (PRS) (Figures 1D–1G and S1A–S1E). From 27 days *in vitro* (DIV) onward, these parameters plateaued, and neuronal network activity remained stable (blue boxes). Because these neuronal networks were generally measured in this stable period (DIV 27–35), we pooled data in this developmental window. In this specific time window, we observed similar patterns of activity and connectivity across all control lines (Figures 1H, S1F–S1O, S2, and S3).

We next determined the specific range of values for each parameter that described the neuronal network phenotype (Figures 1I and S3F; Table S2). Control neuronal networks showed a general level of activity of 3.5 ± 0.2 spike/s, 4.8 ± 0.2 bursts/min, and 3.2 ± 0.1 network bursts/min, with a

Figure 1. Control neuronal networks show a stable phenotype on MEA

(A) Information regarding the ten control lines used in this study. C_6 was recorded on two substrates (H, human laminin; M, mouse laminin). Number of wells represents total number of wells recorded for that line between DIV 27 and 35, including the number of batches. Some batches overlap between lines.

(B) Schematic overview of extracted parameters from MEA (see Table S1).

(C) Representative raster plots of line C_6 showing 60 s of electrophysiological activity across development (DIV 14–42).

(D–G) Neuronal network parameters (of line C_6) develop to reach a certain plateau after DIV 27 (blue box) for (D) MFR, (E) PRS, (F) NBR, and (G) NBD.

(H) Representative raster plots of ten control lines showing 3 min of electrophysiological activity on MEAs.

(I) Graph showing the range in which MEA parameters of all ten control lines behave (mean ±95% confidence interval). Values are first averaged per control line, and then averaged across all control lines.

(J) Percent coefficient of variation explaining the stability of the respective MEA parameter across all ten control lines (mean ± standard deviation of the mean). N = 278 wells (Table S2).

DIV, days *in vitro*; MFR, mean firing rate; PRS, percentage of random spikes; BR, mean burst rate; BD, mean burst duration; BSR, burst spike rate; IBI, inter-burst interval; NBR, network burst rate; NBD, network burst duration; NIBI, network burst IBI; CV_{NIBI}, coefficient of variation of all NIBI's representing the regularity of the NB; RT, rise time; DT, decay time. All means are reported in Table S2.

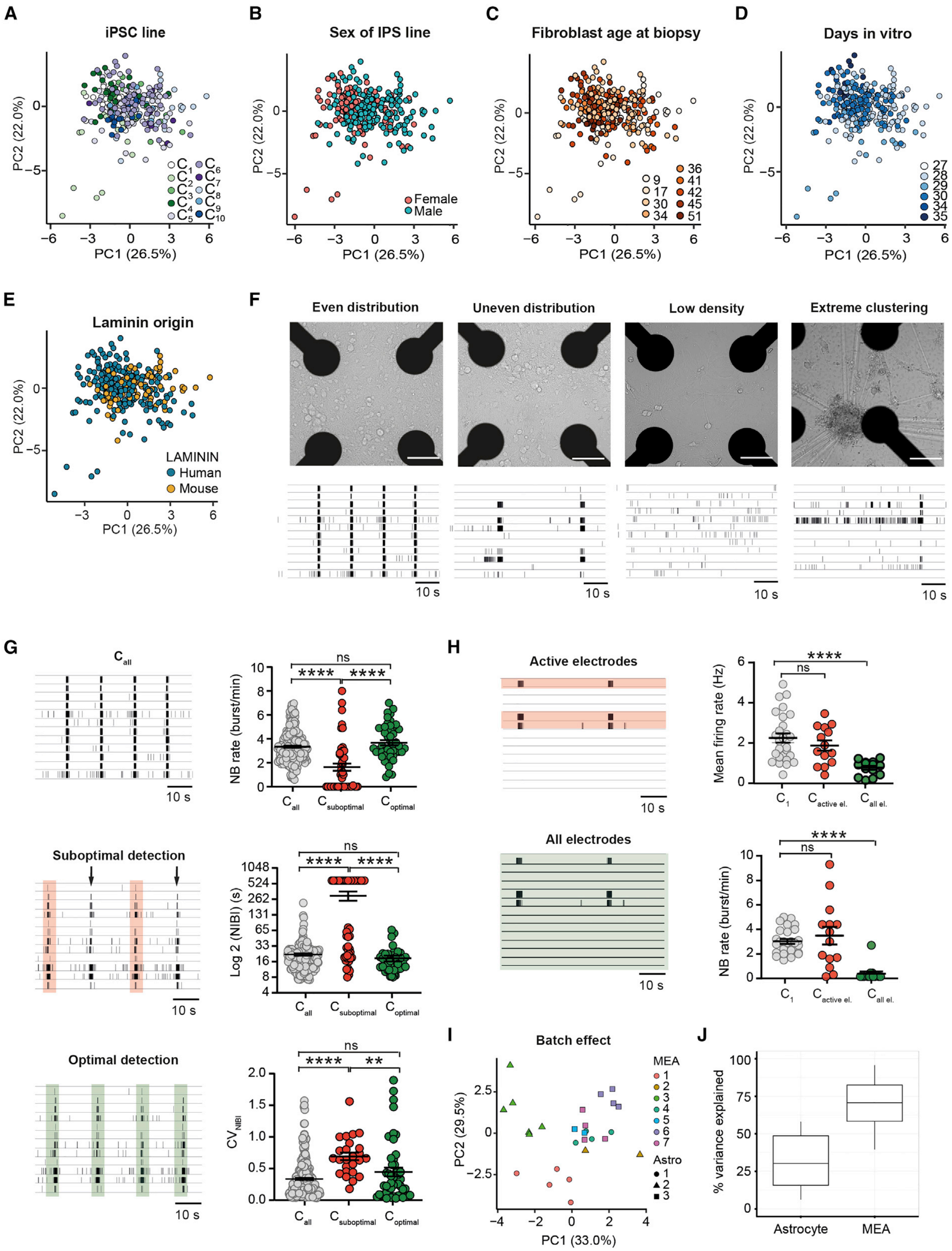

(legend on next page)

duration of 1.28 ± 0.04 s (n_{wells} = 278). We did observe slight differences between individual control lines. For example, control lines C_2 and C_9 exhibited synchronous events at different frequencies compared with the other controls (i.e., 1.4 ± 0.2 and 4.6 ± 0.3 network bursts/min for C_2 and C_9, respectively, Figure S1M), stressing the need of using multiple lines to uncover the full phenotypic spectrum of control neuronal networks. Taken together, these results indicate that neuronal networks on MEA show similar patterns of activity across multiple control lines.

Next, we investigated the variability of the MEA parameters within our control dataset to identify the most robust parameters (i.e., coefficient of variation lower than 50% as cutoff, Figures 1J and S3G). Certain parameters were more stable (i.e., frequency and duration of NBR and NBD, respectively), whereas others were more variable, i.e., MFR, the regularity of the network burst appearance, calculated as the coefficient of variation of the interval distribution between network bursts (CV_{NIBI}), the degree of synchronization (C_0), and link weight. In most of the hiPSC-based MEA studies, the MFR has been used as the main and only parameter, which may confound the characterization and interpretation of the neuronal network behavior. Beside the fact that MFR is one of the most variable parameters reported here, it is highly dependent on cell density (Biffi et al., 2013) and lacks information about network synchronization. Multiple MEA parameters describing both general activity and bursting behavior should be included to obtain a comprehensive characterization of neuronal network behavior.

Confounding factors in experimental design, culturing, and analysis that influence the reliability of neuronal network recordings

Combining all MEA parameters in a principal-component analysis (PCA), we did not observe clear clustering based on hiPSC line (Figure 2A), indicating that there was no consistent line-specific difference at the functional level. To guarantee these reliable neuronal network recordings, we explored which confounding factors introduce variation. Sex and age of the original fibroblast donor had no major effect on the neuronal network phenotype variability (Figures 2B and 2C). Furthermore, we found no clear clustering based on DIV when the cultures reached a stable developmental stage (i.e., DIV 27–35, Figure 2D). However, neuronal networks measured earlier (DIV 14–24) clustered away from measurements performed after DIV 28 (Figure S1P). Thus, pooling data from different developmental stages should be avoided since it likely introduces variation in the data.

Next, we explored whether culturing conditions introduced variation. First, we observed no clear difference between neuronal networks grown on two types of coating (mouse or human laminin) at the stable developmental stage (Figure 2E). However, different developmental trajectories have been observed in neuronal networks grown on mouse and human laminin (Hyysalo et al., 2017), thus pooling and comparison of data from different coatings can affect their comparability. Another culturing variable that could influence network activity is cell distribution. With low-resolution MEA systems (i.e., 12 electrodes

Figure 2. Variables that influence neuronal network phenotype

(A–E) Principal-component analysis (PCA) plot on all parameters showing data of all control lines pooled from DIV 27 to 35 (A) color coded by line, (B) color coded by sex, (C) color coded by the fibroblast age at biopsy, (D) color coded by DIV, and (E) color coded by laminin origin.

(F) Representative images of neuronal cultures grown at different densities and distributions (even, uneven, and low densities, and extreme clustering) and representative raster plots showing 1 min of activity exhibited by neuronal networks in each condition.

(G) Representative raster plots of a well in which the network burst detection was adapted to detect all network burst present. Colored bars represent the detected network burst by software. Comparison of the MEA parameters NBR, NIBI (on log2 scale), and CV_{NIBI} between control pool (C_{all} gray), wells in which not all network burst have been detected ($C_{suboptimal}$, red), and the same wells when optimal detection have been performed ($C_{optimal}$, green) (mean ± standard error of the mean). Kruskal-Wallis ANOVA with Dunn's correction for multiple testing was used to compare between control lines.

(H) Representative raster plots of C1 and a well in which only a few channels are active. In the figure, the electrodes used for analysis are highlighted (green and red for three active electrodes and all electrodes, respectively). Comparison of the MEA parameters MFR and NBR between C1, a well in which the analysis has been performed only on active electrode ($C_{active\ el}$, red) and the same well when the analysis has been performed on all electrodes ($C_{all\ el}$, green) (mean ± standard error of the mean). One-way ANOVA with Tukey correction for multiple testing was used to compare between control lines.

(I) PCA plot on all parameters showing data of one control line (C1) with colors representing MEA batches and shapes representing astrocyte batches.

(J) Percentage of variance explained by astrocyte batch and MEA batch, calculated based on separate linear models to determine the effects of astrocyte and MEA batch independently.

*p = 0.05, **p = 0.01, ***p = 0.001. DIV, days *in vitro*; MFR, mean firing rate; PRS, percentage of random spikes; NBR, network burst rate; NBD, network burst duration; NIBI, network burst IBI; CV_{NIBI}, coefficient of variation of all NIBI's representing the regularity of the NB. All means, p values, and statistic tests used are reported in Table S4.

spaced 300 μm apart), the activity recorded from the electrodes originates from multiple neurons. Therefore, homogeneous distribution of cells on each electrode should be achieved. Indeed, we found that changes in cell density and distribution affected neuronal network functionality (Figure 2F). While an even distribution of neurons on all electrodes was accompanied by synchronous activity involving all channels, an uneven distribution led to events involving only a few channels (Figure 2F, second panel). In addition, neuronal networks with (extreme) low densities exhibited less frequent events (Figure 2F, third panel) or only random spikes (Figure 2F, third panel). Cell clustering led to highly frequent local activity, recorded only by the electrodes close to the cluster (Figure 2F, fourth panel). Thus, cell density and distribution should be consistent to achieve a comparable network pattern, and a density that allows for proper neuron-electrode coupling should be chosen (1,200 cells/mm^2 in this dataset). Neuronal networks with low cell density, clustering, or uneven distribution of cells should be excluded from the analysis.

In addition to culturing conditions, accurate data analysis depended on the selection of proper analysis settings. Suboptimal network burst detection (i.e., not all network bursts were correctly detected) (Figure 2G) sometimes occurred by adhering to the standard settings of the analysis software, or too stringent settings determined by the experimenter. Visually, raster plots of suboptimal detected control networks did not differ from raster plots of the total control pool. However, comparing suboptimal detected control networks with the total control pool resulted in a faulty quantification of the neuronal network organization (Figure 2G, $C_{suboptimal}$ versus C_{all}). When the analysis settings of suboptimal detected networks were changed to more optimal detection settings (i.e., the settings that correctly quantify each network burst, determined by the experimenter's observation for each individual recording), no difference between the two groups was present ($C_{optimal}$ versus C_{all}), as expected from the raster plot. Thus, the experimenter's observation and intervention on data analysis for each recording is essential to obtain accurate results. Similarly, data analysis performed on individual active electrodes led to erroneous results when culturing conditions were not optimal (Figure 2F). When we analyzed only the active electrodes in wells with uneven densities, we obtained similar activity patterns as in wells with an optimal density in which all electrodes were analyzed, resulting in an incorrect representation of the actual neuronal network (Figure 2H, $C_{active\ el.}$ versus C_1). Analysis on all electrodes, however, provided a correct image of the neuronal network phenotype ($C_{all\ el.}$ versus C_1). Thus, stringent criteria should be used when performing data analysis. Control neuronal networks should display at least certain activity levels to be included in further analysis, including an MFR > 0.1 spike/s, a BR > 0.4 bursts/min, and an NBR > 1 network burst/min, and synchronous activity should be observed in most of the channels. General activity (i.e., spikes) should be detected in at least 80% of the electrodes and analysis should be performed on all electrodes rather than only on the active ones.

Finally, we investigated the effect of both independent astrocyte batches and MEA batches (i.e., independent neuronal preparations on MEA) on the neuronal network behavior. PCA showed that samples cluster based on astrocyte and MEA batch (Figure 2I), indicating that different batches affected the neuronal network phenotype. We calculated the percentage of variance explained by astrocyte batch and MEA batch separately. On all MEA parameters combined, astrocyte batch explained 32% of the variation and MEA batch explained 69% of the variation (Figure 2J). The PRS, burst spike rate (BSR), BD, NBD, and decay time (DT) were significantly affected by both astrocyte batch and MEA batch (adj. $p < 0.05$) (Table S4). In addition, MEA batch significantly affected the BR, NBR, network inter-burst interval (NIBI), and rise time (RT) (adj. $p < 0.05$) (Table S4). These results stress the need for using multiple experimental batches when comparing different lines to correct for this technical variation (i.e., at least two MEA batches, preferentially with astrocytes belonging to the same batch).

In summary, our results indicate that certain standards should be followed to ensure that reliable data were obtained from MEA experiments (Table 1). To generate reproducible neuronal control network phenotypes, one needs to (1) culture sufficient neurons that are homogeneously distributed, (2) properly select the detection settings, (3) pool data only in a certain developmental time window, and (4) use sufficient experimental batches.

The MEA system is a reliable platform for disease phenotyping

To confirm that control neuronal networks can be used as a platform for disease phenotyping, we compared patient neuronal network activity from two neurodevelopmental disorders (NDD) with the total control pool. In particular, we re-analyzed our previously published data from three patients with MELAS syndrome (two females and one male, mean age 34.7 years) (Klein Gunnewiek et al., 2020) and four patients with KS (three females and one male, mean age 27.5 years) (Frega et al., 2019) (Figure 3A). Since control neuronal networks were stable between DIV 27 and 35, recordings from MELAS patient lines (n_{wells} = 112), as well as KS patient lines (n_{wells} = 58), were pooled in the same time window.

Neuronal networks from MELAS patients showed a different network phenotype compared with the control

Table 1. List of recommendations

Experimental design	12 wells per condition, divided over 2 MEA batches	page 9, paragraph 1
	comparison of at least 3 control or patient lines	page 9
	inclusion of isogenic patient-control set	page 7
Cell culturing	homogeneous distribution of cells	page 6, paragraph 1 Figure 2F
	cell density allowing neuron-electrode coupling (i.e., 1,200 cells/mm^2)	page 6, paragraph 1
	same astrocyte batch for conditions under comparison	page 6, paragraph 2
Data analysis	analyze multiple MEA parameters	page 6, 7, 9
	pooling of data only in similar developmental stages	page 5, paragraph 2 Figure S1H
	in control: MFR > 0.1 spike/s, BR > 0.4 bursts/min, NBR > 1 NB/min, active channels > 80%, channels in NB > 25%	page 6
	analysis on all electrodes	page 6, paragraph 1 Figure 2H

Recommendations are provided regarding experimental design, cell culture conditions, and MEA data analysis. Page numbers, including paragraph numbers are shown, which refer to sections of the text that provide information and data to support our recommendations.

pool (Figure 3B). In line with previous findings (Klein Gunnewiek et al., 2020), the phenotype was mainly driven by a strong reduction in level of spiking and network bursting activity, together with an increased PRS (Figures 3C–3F). In addition to previously published data, MELAS neuronal networks exhibited bursts with a shorter duration compared with the control pool (Figure 3E). We did not observe any difference in burst shape, RT, or DT of MELAS patient network bursts (Figure S4A). Neuronal networks derived from MELAS patients did show a lower level of correlation (C_{peak}) and synchronization (C_0) in all channels (Figure S4B). Furthermore, despite a comparable number of functional connections among electrodes in control and MELAS patient neuronal networks, we observed that the connections between MELAS neurons were weaker (Figure S4C). PCA confirmed that MELAS patient networks clustered separately from controls (Figure 3L).

Next, we compared neuronal networks from patients with KS with our total control pool and uncovered a significantly different network phenotype (Figure 3B). In line with previously published findings (Frega et al., 2019), the KS phenotype was mainly characterized by a lower frequency of (network) bursts with a longer duration (Figures 3G–3I). In addition, KS neuronal networks exhibited a different network burst shape and an increased DT (Figures 3J and 3K), and showed a lower level of synchronicity and correlation and weaker connections between neurons compared with controls (Figures S4D and S4E). We observed that the differences between patients and controls were more pronounced in KS_4 as compared with the other KS lines (Figure S4F). PCA confirmed that KS neuronal networks clustered away from controls based on these parameters (Figure 3M).

In conclusion, the neuronal network phenotypes of MELAS and KS lines differed from controls on distinct parameters. Indeed, MELAS and KS samples cluster away from controls, but also clearly cluster away from each other (Figure 3N). The ability to distinguish two NDDs based on their neuronal network phenotypes demonstrates that the MEA system is an adequate platform for disease-specific phenotyping.

Comparing patients with isogenic controls reveals a more detailed phenotype

Isogenic hiPSC lines are increasingly used to improve identification of genotype-phenotype correlations. We compared data from three MELAS mosaic patient-control isogenic sets, one KS mosaic patient-control set and one KS CRISPR-Cas9-engineered isogenic set. The difference between each MELAS and KS isogenic set was explained by the same parameters as when patient lines were compared with all control lines (Figures 3 and 4). We also found that the difference between patient and control lines was larger for isogenic sets compared with all lines combined, as indicated by the higher variance explained by disease status (Figures 3L, 3M, 4G, and 4N).

A

Name	Gender	Age	Genotype	Isogenic control	# wells/batches	DIVs pooled	Coating
KS1	F	13	c.3181–80_3233del	-	15/3	29	Mouse
KS2	F	12	c.3125G > A	-	15/2	29	Mouse
KS3	F	34	Heterozygous 233 kbp deletion of chromosome 9	C9	16/2	29	Mouse
KS4	M	51	Heterozygous *EHMT1* mutation in Exon 2	C10	12/2	29	Mouse
M1	F	17	m.3243A > G 66% HP	C2	24/5	28,29	Human
M2	F	45	m.3243A > G 65% HP	C4	23/4	29-35	Human
M3	M	42	m.3243A > G 61% HP	C5	68/17	28-34	Human
	M:F=2:5				173/32		H:M=4:3

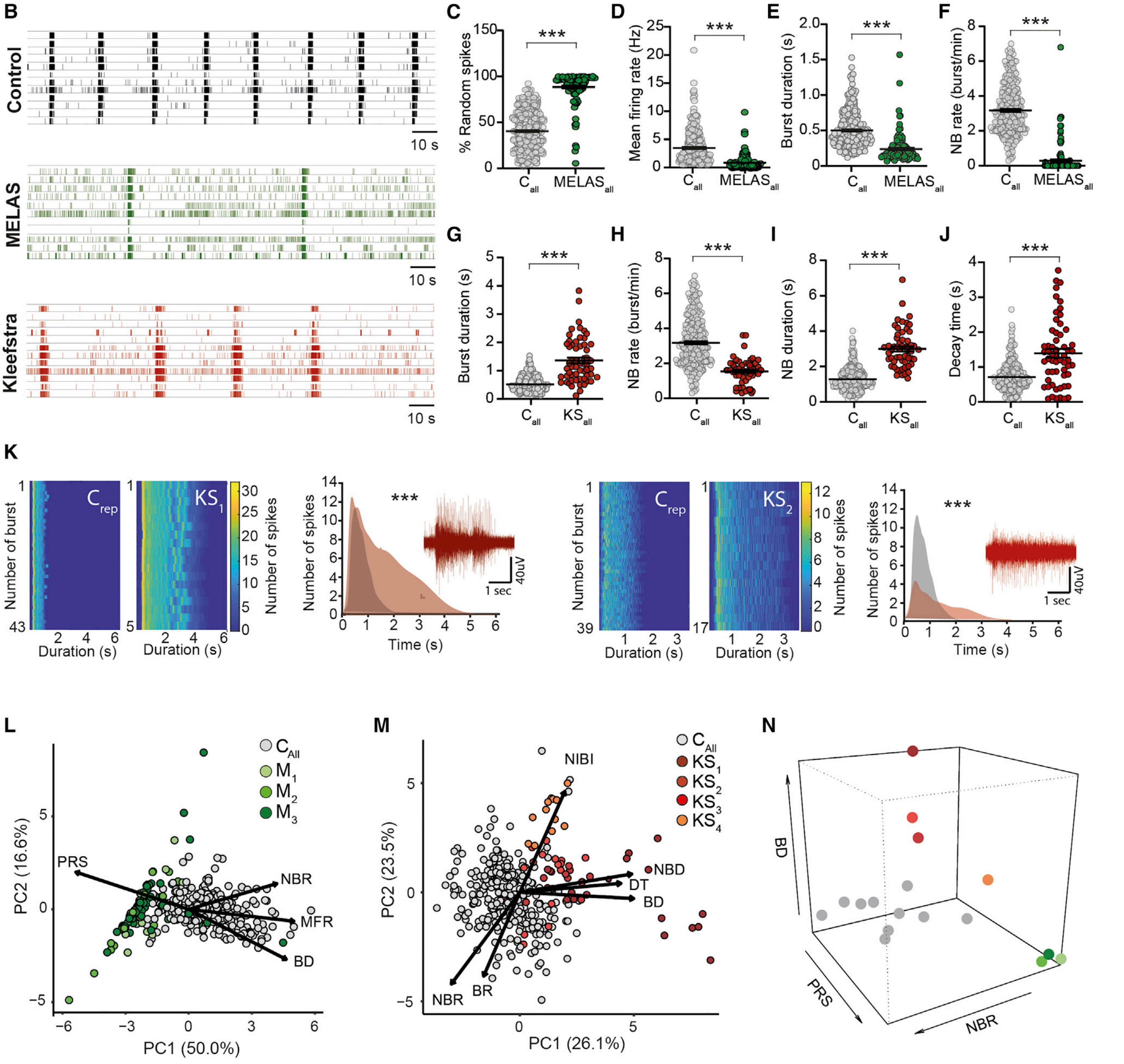

(legend on next page)

For some parameters, we observed smaller differences in one MELAS set (i.e., isogenic set 1) as compared with the other isogenic sets (Figures 4A–4E). This was mainly driven by a difference in the isogenic control (C_2) compared with all controls, rather than a less pronounced MELAS phenotype (Figures S1F–S1O). While comparing isogenic sets, we found a significant difference in the network burst shape of two MELAS isogenic patient-control sets (Figure 4F), a phenotype that was not distinguished when comparing MELAS patients to control pool. Some line-specific differences were also found in the MEA parameters, explaining the KS network phenotype. Whereas the DT was not affected in KS_3 compared with its isogenic control, and only a trend was observed in network burst duration for KS_4, these parameters were altered in all other KS lines (Figures 4K–4M, and S4G). Post hoc power calculation revealed that the parameters that explained the MELAS and KS phenotypes reached a power higher than 0.95 (Table S3), demonstrating the validity of our results. When performing an a priori power calculation on each patient-control isogenic set or all controls compared with all patient lines, we found that a minimum of 12 wells per line should be included in the analysis to observe a patient phenotype on multiple MEA parameters (Table S3).

To conclude, disease phenotypes are generally consistent between different lines from patients with the same disorder, even though some line-specific differences can be observed. This persisted when MELAS and KS lines were compared with their corresponding isogenic controls, highlighting the importance of using multiple patient lines to uncover the full phenotypic spectrum. Nevertheless, we show that isogenic patient-control sets uncover more pronounced phenotypes, emphasizing the advantage of isogenic sets.

DISCUSSION

Despite the increasing popularity of MEAs for disease phenotyping of hiPSC-derived neuronal networks, there is little insight into the variability of control networks and which conditions influence this. Here, we performed a meta-analysis of, to our knowledge, the largest dataset of hiPSC-derived *Ngn2*-induced excitatory neuronal networks on MEA, to describe a standard for control network signatures. We uncovered that neuronal networks derived from ten different healthy subjects clustered together in PCA, regardless of whether they were cultured by different researchers over the course of years, and independent of sex and age at fibroblast biopsy.

These control neuronal networks were very comparable because we adhered to a strict set of guidelines (Table 1). First, networks could only be pooled in the time window between DIV 27 and 35, as networks generated by *Ngn2* overexpression in our lab presented stable activity at this stage. Many factors can influence the timing of this stable network activity. For example, *Ngn2*-neurons mature significantly faster than neurons generated using small-molecule supplementation protocols (Mertens et al., 2016). In addition, while neuronal networks grown on human or mouse laminin showed no difference after DIV 28, cultures grown on human laminin can mature slower (Hyysalo et al., 2017). Therefore, one must define the stable developmental period depending on each protocol, before pooling and comparing data.

Second, as hiPSC culture practices and differentiation protocols consist of many steps, small differences in handling cells can accumulate over time into different outcomes (Volpato and Webber, 2020). We showed that astrocyte and MEA batch introduced variability, and advise to

Figure 3. MEAs pose a reliable platform for genotype-phenotype correlations
(A) Information regarding the seven patient lines included in this study. Isogenic controls represent the lines made from the same founder somatic cell line. Number of wells represents total number of wells recorded for that line between DIV 27 and 35, including the number of batches. Some batches overlap between lines.
(B) Representative raster plots showing 3 min of electrophysiological activity from control, MELAS, and KS patient lines.
(C–F) Graphs showing the values of four MEA parameters, including (C) PRS, (D) MFR, (E) BD, and (F) NBR for control and MELAS neuronal networks (mean ± standard error of the mean).
(G–J) Graphs showing four MEAs, including (G) BD, (H) NBR, (I) NBD, and (J) DT for control and KS neuronal networks (mean ± standard error of the mean). Mann-Whitney U test with Bonferroni correction for multiple testing was used to compare between patient lines and their isogenic controls (Table S4).
(K) Representative network burst alignment from one recording of a representative control and KS1, and a representative control and KS2. Inset: extracted burst shape and representative raw trace of a network burst (sample size for C representative: C_6, n = 58, C_9, n = 12, KS_1, n = 15, KS_2, n = 15, multiple t test on bins using the Holm-Sidak method, p < 0.0001 for both comparisons).
(L) PCA plot on 7 MEA parameters, showing parameters that explain the differences in network behavior between C_{all} (278 wells from 10 control lines) and M1-3.
(M) PCA plot on 12 MEA parameters, showing parameters that explain the differences in network behavior between C_{all} and KS1-4.
(N) 3D scatterplot showing PRS, BD, and NBR for all MELAS (green), KS (red), and control lines (gray).
**p = 0.01 and ***p = 0.001. DIV, days *in vitro*; MFR, mean firing rate; PRS, percentage of random spikes; BD, mean burst duration; NBR, network burst rate; NBD, network burst duration; DT, decay time. All means, p values, and statistic tests used are reported in Table S4.

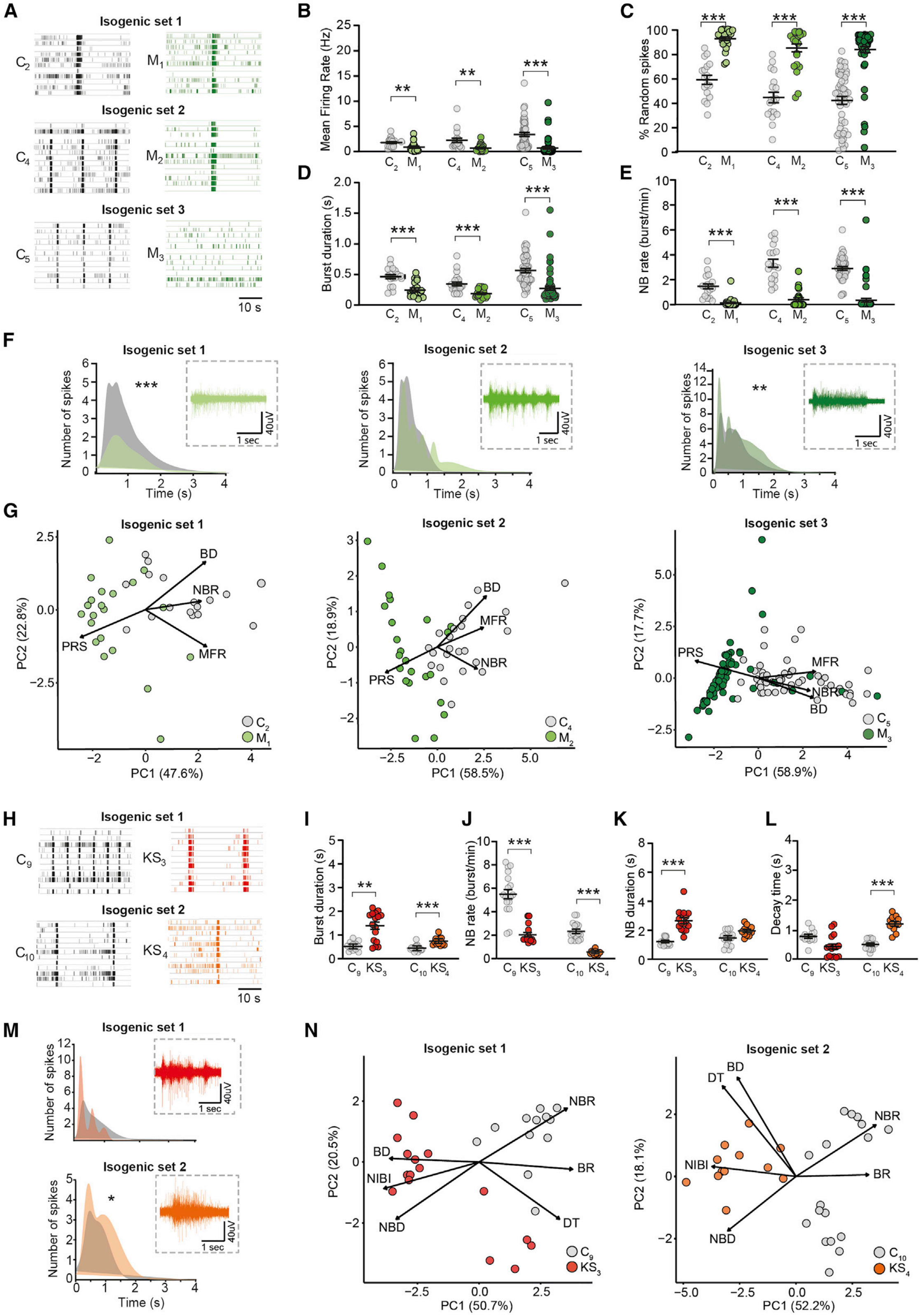

(legend on next page)

use at least two MEA batches with the same astrocyte batch. As an exception, one MEA batch can be used in drug-screening assays, when cell line variability is accounted for by comparing interventions. In addition, we advise to exclude wells with low or uneven cell density and critically look at the density and distribution on the electrode grid in conjunction with the corresponding data.

Third, it is essential to accurately analyze the data and include multiple parameters that describe the network activity. The choice of the analysis settings for data extraction can largely influence the results. Indeed, we showed that these settings need to be fine-tuned, depending on the observation of the experimenter to accurately detect different phenotypic signatures. Network bursts exhibited by patient-derived neuronal networks might be incorrectly detected with commonly used settings, since these were conventionally chosen based on network bursts in control networks. Moreover, it is possible that the observed phenotype cannot be described using any of the commonly used parameters, and new parameters should be introduced to capture these signatures. Indeed, we showed that the extraction of additional parameters from MEA data revealed previously unseen phenotypes.

Finally, we determined the MEA parameter variability, since stable parameters are the most trustworthy to identify a disease phenotype. Interestingly, our data show that the MFR is one of the most variable parameters. In addition, it only describes the general level of activity and is largely dependent on cell density. The MFR should therefore be interpreted with caution when solely used to describe a phenotype, while this is a common practice in the literature, as it is easily extracted from the data (Chailangkarn et al., 2017; Lu et al., 2016; Wainger et al., 2014). Other variable parameters that we determined here (IBI, CV_{NIBI}, RT, C_0, and link weight), can also be linked to alterations in cell density. To use MEA parameters with high variability to determine a patient phenotype, one should include multiple supporting MEA parameters to describe the neuronal network characteristics.

We found a strong segregation between control and MELAS and KS neuronal networks. More interestingly, we found that KS and MELAS neuronal networks were distinguished by different MEA parameters, indicating the potential of MEA recordings to distinguish two different NDD phenotypes. Previous literature showed that neuronal networks with different mutations associated with the same NDD depicted a similar phenotype, albeit characterized by an individual set of parameters. For example, we previously identified that rat cortical networks deficient for the KS spectrum genes *Ehmt1*, *Mll3*, *Mbd5*, and *Smarcb1* all displayed hyperactive neuronal networks. However, whereas EHMT1- and SMARCB1-deficient networks showed a significantly higher MFR, MLL3-deficient networks showed a higher NBR (Frega et al., 2020). Likewise, a recent study that investigated *Ngn2*-induced neuronal network behavior of 12 autism spectrum disorder patients revealed hyperactive neuronal networks specifically from a patient with *CNTN5* and a CRISPR-Cas9-engineered line with an *EHMT2* mutation (Deneault et al., 2019). Together, this strengthens the evidence that early disease-associated network phenotypes can be revealed using MEAs, and that hiPSC-derived neurons are a powerful model to study genotype-phenotype correlations.

Although the phenotype of control neuronal networks was robust, it must be noted that we still observed significant variation between individual control lines, and a similar variation was observed in the patient neuronal network phenotype. While we cannot rule out a

Figure 4. Characterization of isogenic control and patient networks

(A) Representative raster plots showing 1 min of activity from three control (C_2, C_4, C_5)-MELAS ($M_{1\text{-}3}$) isogenic sets.

(B–E) Comparison of the MEA parameters (B) MFR, (C) PRS, (D) BD, and (E) NBR for each corresponding MELAS isogenic patient-control set (mean ± standard error of the mean).

(F) Burst shape and representative raw trace of a network burst from C_2 and M_1, C_4 and M_2, and C_5 and M_3 (sample size for C_2, n = 15, C_4, n = 23, C_5, n = 55, M_1, n = 22, M_2, n = 8, and M_3, n = 7, multiple t test on bins using the Holm-Sidak method, C_2 versus M_1, $p < 0.001$; C_5 versus M_3, p = 0.00021) (Table S4).

(G) PCA plots on seven7 MEA parameters for MELAS isogenic patient-control sets C2 and M1, C4 and M2, and C5 and M3 showing MEA parameters affected in MELAS.

(H) Representative raster plots showing 1 min of activity from two control ($C_{9\text{-}10}$)-KS patient ($KS_{3\text{-}4}$) isogenic sets.

(I–L) Comparison of the MEA parameters (I) BD, (J) NBR, (K) NBD, and (L) DT for each corresponding KS isogenic patient-control set (mean ± standard error of the mean).

(M) Burst shape and representative raw trace of a network burst from C_9 and KS_1, C_{10} and KS_4 (sample size for C_9, n = 12, C_{10}, n = 17, KS_3, n = 16, and KS_4, n = 12, multiple t test on bins using the Holm-Sidak method) (Table S4).

(N) PCA plot on 12 MEA parameters for KS isogenic patient-control sets C_9 and KS_3 and C_{10} and KS_4 showing MEA parameters affected in KS. *p = 0.05, **p = 0.01, ***p = 0.001. DIV, days *in vitro*; MFR, mean firing rate; PRS, percentage of random spikes; BR, mean burst rate; BD, mean burst duration; BSR, burst spike rate; IBI, inter-burst interval; NBR, network burst rate; NBD, network burst duration; NIBI, network burst IBI; CV_{NIBI}, coefficient of variation of all NIBI's representing the regularity of the NB; RT, rise time; DT, decay time. All means, p values, and statistic tests used are reported in Table S4.

patient-specific component, this variation likely reflects normal variation in the general population (Germain and Testa, 2017). Gene expression and DNA methylation profiles vary significantly among hiPSC lines, of which common genetic variation is the main driver (DeBoever et al., 2017; Germain and Testa, 2017; Kilpinen et al., 2017). Indeed, previous literature uncovered that the heterogeneity within 25 different hiPSC lines on a transcriptional level was due to differences in genetic background (Rouhani et al., 2014). Adding to this, the differentiation efficiency of hiPSC-derived neurons can also contribute to variation seen between lines (Hu et al., 2010). We speculate that this difference in common genetic variation and differentiation efficiency can result in small variations on a functional level. To correct for line-specific differences and variability, multiple lines from different individuals should be used to determine the patient neuronal network phenotype.

In summary, we here provide a set of guidelines to reduce the variability in neuronal network recordings on MEAs (Table 1). We expect that, if cultures are handled according to these guidelines, our control dataset can be used as a reference database to determine the performance of *Ngn2*-induced control lines. An extensive list of literature has shown that network parameters can differ between different sources, neuronal differentiation protocols, or species (Heikkilä et al., 2009; Hyysalo et al., 2017; Napoli and Obeid, 2016; Odawara et al., 2016). While we expect that other neuronal model systems will show network parameters in a different range than reported here, the guidelines that we propose can nevertheless be generalized. Following these guidelines, MEAs are a valuable tool to describe the neuronal network phenotypes in hiPSC-derived neuronal networks.

EXPERIMENTAL PROCEDURES

hiPSC line origin and generation

All hiPSC lines used to generate this dataset were obtained by reprogrammed skin fibroblasts. We used ten hiPSC control lines in total, of which five are independent control lines (C_1, $C_{6\text{-}8}$, and C_{10}). To illustrate that the model that we use is stable enough to uncover patient-specific phenotypes, we included both KS patient and MELAS patient lines, as well as isogenic patient-control sets. For KS, we included two isogenic sets consisting of C_9 and KS_3 and C_{10} and KS_4, which have been described previously in detail (Frega et al., 2019). In addition, we included two KS patient hiPSC lines, KS_1 and KS_2, which were previously characterized and derived from a 13-year-old female and a 12-year-old female, respectively, diagnosed with KS (Frega et al., 2019). For MELAS, we included three isogenic sets generated from MELAS individuals with different levels of m.3242A > G heteroplasmy (0% or ±60%), consisting of C_2 and M_1, C_4 and M_2, and C_5 and M_3, which have been described previously in detail (Klein Gunnewiek et al., 2020). All generated hiPSC clones were tested for pluripotency markers (OCT3/4, SOX2, and NANOG) using immunocytochemistry and qPCR. A detailed description of all hiPSC lines included in this study can be found in the supplemental information.

hiPSCs were cultured on E8 Flex basal medium (Thermo Fisher Scientific, no. A2858501) supplemented with primocin (0.1 μg/mL, Invivogen, no. ant-pm-1), puromycin (0.5 μg/mL) (Sigma-Aldrich, no. P9620) and G418 (50 μg/mL) (Sigma-Aldrich, no. A1720) at 37°C/5% CO_2, on either human recombinant laminin LN521 (Biolamina, no. LN521-02) or Matrigel (Corning, no. 356237)-coated plates. Medium was refreshed every 2 days and cells were passaged approximately every 3 days using ReLeSR (STEMCELL Technologies, no. 05873), an enzyme-free passaging reagent.

Neuronal differentiation and culture

hiPSCs were differentiated into upper layer excitatory cortical neurons by doxycycline-inducible expression of the neuronal transcription factor neurogenin 2 (*Ngn2*) (Zhang et al., 2013), according to a previously published protocol (Frega et al., 2017). To generate single cells, *rtTA/Ngn2*-positive hiPSCs were detached by incubating accutase (Sigma-Aldrich, no. A6964) at 37°C/5%CO_2 and resuspended in E8 basal medium (Thermo Fisher, no. A15170-01), supplemented with primocin (0.1 μg/mL), RevitaCell (Thermo Fisher, no. A2644501) (10 μg/mL), and doxycycline (Sigma-Aldrich, no. D9891) (4 μg/mL) to induce TetO gene expression. Cells were plated at a density of 20,000 cells per MEA well (600 neurons/mm^2), which were pre-coated with poly-L-ornithine hydrobromide (Sigma-Aldrich, no. P3655-10MG) (50 mg/mL) and, depending on experiment, either human recombinant laminin LN521 (5 mg/mL) or laminin from Engelbreth-Holm-Swarm murine sarcoma basement membrane ([mouse laminin], Sigma-Aldrich, no. L2020) (20 μg/mL). At DIV 1, the medium was changed using filtered DMEM/F12 supplemented with primocin (0.1 μg/mL), doxycycline (4 μg/mL), 1% N-2 supplement (Thermo Fisher, no. 17502-048), 1% MEM non-essential amino acid solution (Sigma-Aldrich, no. M7145), neurotrophin-3 ([NT3] Promokine no. C-66425) (10 ng/mL), recombinant human brain-derived neurotrophic factor ([BDNF] Promokine, no. C-66212) (10 ng/mL), and mouse laminin (0.2 μg/mL). At DIV 2, rat embryonic astrocytes were added in a 1:1 ratio to support neuronal maturation and viability (Frega et al., 2017). The medium was changed at DIV 3 to filtered neurobasal medium (Thermo Fisher, no. 21103-049) supplemented with primocin (0.1 μg/mL), B-27 (Thermo Fisher, no. 17504044) (20 μg/mL), GlutaMAX (Thermo Fisher, no. 35050061) (10 μg/mL), doxycycline (4 μg/mL), NT3 (10 ng/mL), BDNF (10 ng/mL), and cytosine β-D-arabinofuranoside (Sigma-Aldrich, no. C1768) (2 μM), to remove proliferating cells from the culture. From DIV 5 to 9, 50% of the neurobasal medium supplemented with B-27, GlutaMAX, Pen/Strep, doxycycline, NT3, and BDNF, was refreshed every 2 days. From DIV 9 to 21 onward the neurobasal medium was, in addition, supplemented with 2.5% fetal bovine serum (Sigma-Aldrich, no. F7524) to support astrocyte viability. All neuronal cultures were kept in incubation at 37°C/5%CO_2. Control lines C1, C6, and C7 were partly cultured in the absence of doxycycline from DIV 13 onward. No significant effect between wells cultured with and without doxycycline were

found, therefore all data for these respective lines is pooled (data not shown).

MEA recordings and data analysis

To record spontaneous network activity, multiwell MEAs were used that consisted of 24 individual wells (Multichannel Systems, MCS GmbH, Reutlingen, Germany). Each well was embedded with 12 electrodes with a diameter of 30 μm, spaced 300 μm apart. The activity of neuronal networks growing on MEAs was recorded for 10 min (after a 10 min acclimatization period) in a recording chamber that was maintained at 37°C/95% O_2/5% CO_2. The raw signal was sampled at 10 kHz and filtered with a high-pass second-order Butterworth filter with a 100 Hz cutoff frequency and a low-pass fourth-order Butterworth filter with a 3,500 Hz cutoff frequency. The noise threshold for individual spike detection was set at ±4.5 standard deviations.

Data analysis

Offline data analysis was performed using Multiwell-Analyzer software (Multichannel Systems) that permitted the extraction of spike-trains, and either a custom-made in-house code developed in MATLAB (MathWorks, Natick, MA, USA) or a software package called SPYCODE (Bologna et al., 2010; Frega et al., 2017), which both allowed the extraction of parameters describing the spontaneous network activity. A detailed description of the acquisition of different MEA parameters can be found in the supplemental information.

To guarantee sufficient experimental replicates, we included experiments with a minimum of 12 wells per hiPSC line measured across at least two independent batches. Control neuronal networks showing an MFR < 0.1 Hz and BR < 0.4 bursts/min were excluded from analysis. Wells were excluded from analysis if they did not have network bursts at DIV 27. Furthermore, wells that displayed insufficient quality, for example, a low density of cells or cell clumping, were discarded. All experiments, excluding experiments where we investigated neuronal network development over time, were carried out during a 1-week time interval, spanning DIV 27 to 35. Since our results, and previous research, has shown that network burst parameters are stable from DIV 27 onward, data from DIV 27 to 35 were pooled (Frega et al., 2019). When analyzing multiple developmental time points of one MEA batch, we determined the network burst detection settings at the latest DIV and kept these settings throughout the analysis, working our way backward to the earliest DIV. Wells in which a reduction of network parameters was observed were also excluded.

Statistical analysis

Data were analyzed using Prism GraphPad 8 (GraphPad Software, CA, USA). We ensured normal distribution using a Kolmogorov-Smirnov normality test. To determine statistical significance, p values < 0.05 were considered to be significant. Statistical analysis on all control lines in Figures S1F–S1O, S2B, S2C, S3B–S3E, S4F, and S4G (for KS1 and KS2) were performed using a Kruskal-Wallis ANOVA with post hoc Dunn's correction for multiple testing or one-way ANOVA with Tukey correction or multiple testing depending on the distribution of the data. When comparing means of two variables at one individual time point we analyzed significance between groups by means of a Mann-Whitney U test (Figures 3C–3J, 4B–4E, 4I–4L, and S4F) (for KS3 and KS4), and, if applicable, corrected post hoc for multiple testing using the Bonferroni method. Statistics on histograms was performed using multiple t test on bins using the Holm-Sidak method (Figures 3K, 4F, and 4M). Data are presented as mean ± standard error of the mean (SEM) if not differently specified. Means and p values are reported in Table S4. To check the variability in the dataset we calculated the coefficient of variation on each parameter independently for all control lines (Figures 1J and S3G; Table S2).

Data visualization

PCA was performed on various MEA parameters using the prcomp function from stats R package (v.3.6.1.) on standardized (*Z* score scaled) data. PCA figures were generated using the ggplot function from the ggplot2 R package (v.3.2.1). A detailed description of the analysis per PCA plot can be found in the supplemental information. A 3D scatterplot was made for all control (n_{wells} = 278, N_{plates} = 47), Kleefstra (n_{wells} = 58, N_{plates} = 9), and MELAS (n_{wells} = 112, N_{plates} = 23) samples together showing PRS, BD, and NBR using the scatter3d function from scatterplot3d R package (v.0.3-41).

Animals

The rodent astrocytes used in this study were derived from embryonic day 18 rat brains, as described previously (Frega et al., 2017; Mccarthy, 1980). Animal experiments were conducted in conformity with the Animal Care Committee of the Radboud University Nijmegen Medical Center, the Netherlands, and conform to the guidelines of the Dutch Council for Animal Care and the European Communities Council Directive 2010/63/EU.

Data and code availability

Exports of the raw data (i.e., Peak Trains, which are.mat files containing the timing and amplitude of each detected spike for all electrodes in one MEA) from all recorded patient and control MEAs and all codes used in this manuscript have been deposited on Mendeley data with https://doi.org/10.17632/bvt5swtc5h.1.

SUPPLEMENTAL INFORMATION

Supplemental information can be found online at https://doi.org/10.1016/j.stemcr.2021.07.001.

AUTHOR CONTRIBUTIONS

M.F. and N.N.K. conceived and supervised the study. B.M., A.H.A.V., E.J.H.v.H., T.M.K.G., K.L., C.S., and M.F. performed all experiments. B.M., A.H.A.V., E.J.H.v.H., G.P., and M.F. performed data analysis. T. Kleefstra, T. Kozicz, H.v.B., D.S., N.N.K., and M.F. provided resources, conceptualization, and intellectual content. B.M., A.H.A.V., E.J.H.v.H., and M.F. wrote the paper. T.M.K.G., G.P., K.L., C.S., T. Kleefstra, T. Kozicz, H.v.B., D.S., and N.N.K. edited the paper.

ACKNOWLEDGMENTS

This work was supported by grants from: the Netherlands Organisation for Scientific Research, NWO-CAS grant 012.200.001 (to

N.N.K.); the Netherlands Organisation for Health Research and Development ZonMw grant 91217055 (to. H.v.B. and N.N.K.); SFARI grant 610264 (to N.N.K.); ERA-NET NEURON-102 SYN-SCHIZ grant (NWO) 013-17-003 4538 (to D.S.) and ERA-NET NEURON DECODE! grant (NWO) 013.18.001 (to N.N.K.), the Dutch epilepsiefonds WAR 18-02 (to N.N.K.).

Received: January 12, 2021
Revised: June 30, 2021
Accepted: July 1, 2021
Published: July 29, 2021

REFERENCES

Biffi, E., Regalia, G., Menegon, A., Ferrigno, G., and Pedrocchi, A. (2013). The influence of neuronal density and maturation on network activity of hippocampal cell cultures: a methodological study. PLoS One *8*. https://doi.org/10.1371/journal.pone.0083899.

Bologna, L.L., Pasquale, V., Garofalo, M., Gandolfo, M., Baljon, P.L., Maccione, A., Martinoia, S., and Chiappalone, M. (2010). Investigating neuronal activity by SPYCODE multi-channel data analyzer. Neural Netw. *23*, 685–697. https://doi.org/10.1016/j.neunet.2010.05.002.

Chailangkarn T., Trujillo C.A.., Freitas B.C.., Hrvoj-mihic B.., Herai R.H., Yu D.X., Brown T.T., Marchetto M.C.N., Mchenry L., Stefanacci L.., et al. (2017). HHS public access. 536, 338–343. https://doi.org/10.1038/nature19067.A

DeBoever, C., Li, H., Jakubosky, D., Benaglio, P., Reyna, J., Olson, K.M., Huang, H., Biggs, W., Sandoval, E., D'Antonio, M., et al. (2017). Large-scale profiling reveals the influence of genetic variation on gene expression in human induced pluripotent stem cells. Cell Stem Cell *20*, 533–546.e7. https://doi.org/10.1016/j.stem.2017.03.009.

Deneault, E., Faheem, M., White, S.H., Rodrigues, D.C., Sun, S., Wei, W., Piekna, A., Thompson, T., Howe, J.L., Chalil, L., et al. (2019). CNTN5 –/+ or EHMT2 –/+ human iPSC-derived neurons from individuals with autism develop hyperactive neuronal networks. eLife *8*, 1–26. https://doi.org/10.7554/eLife.40092.

Engle, S.J., Blaha, L., and Kleiman, R.J. (2018). Best practices for translational disease modeling using human iPSC-derived neurons. Neuron *100*, 783–797. https://doi.org/10.1016/j.neuron.2018.10.033.

Frega, M., Linda, K., Keller, J.M., Gümüş-Akay, G., Mossink, B., van Rhijn, J.R., Negwer, M., Klein Gunnewiek, T., Foreman, K., Kompier, N., et al. (2019). Neuronal network dysfunction in a model for Kleefstra syndrome mediated by enhanced NMDAR signaling. Nat. Commun. *10*, 1–15. https://doi.org/10.1038/s41467-019-12947-3.

Frega, M., Selten, M., Mossink, B., Keller, J.M., Linda, K., Moerschen, R., Qu, J., Koerner, P., Jansen, S., Oudakker, A., et al. (2020). Distinct pathogenic genes causing intellectual disability and autism exhibit a common neuronal network hyperactivity phenotype. Cell Rep. *30*, 173–186.e6. https://doi.org/10.1016/j.celrep.2019.12.002.

Frega, M., van Gestel, S.H.C., Linda, K., van der Raadt, J., Keller, J., Van Rhijn, J.-R., Schubert, D., Albers, C.A., and Nadif Kasri, N. (2017). Rapid neuronal differentiation of induced pluripotent stem cells for measuring network activity on micro-electrode arrays. J. Vis. Exp. *119*, 1–10. https://doi.org/10.3791/54900.

Fukushima, K., Miura, Y., Sawada, K., Yamazaki, K., and Ito, M. (2016). Establishment of a human neuronal network assessment system by using a human neuron/astrocyte co-culture derived from fetal neural stem/progenitor cells. J. Biomol. Screen. *21*, 54–64. https://doi.org/10.1177/1087057115610055.

Germain, P.L., and Testa, G. (2017). Taming human genetic variability: transcriptomic meta-analysis guides the experimental design and interpretation of iPSC-based disease modeling. Stem Cell Reports *8*, 1784–1796. https://doi.org/10.1016/j.stemcr.2017.05.012.

Heikkilä, T.J., Ylä-Outinen, L., Tanskanen, J.M.A., Lappalainen, R.S., Skottman, H., Suuronen, R., Mikkonen, J.E., Hyttinen, J.A.K., and Narkilahti, S. (2009). Human embryonic stem cell-derived neuronal cells form spontaneously active neuronal networks in vitro. Exp. Neurol. *218*, 109–116. https://doi.org/10.1016/j.expneurol.2009.04.011.

Hu, B.Y., Weick, J.P., Yu, J., Ma, L.X., Zhang, X.Q., Thomson, J.A., and Zhang, S.C. (2010). Neural differentiation of human induced pluripotent stem cells follows developmental principles but with variable potency. Proc. Natl. Acad. Sci. U S A *107*, 4335–4340. https://doi.org/10.1073/pnas.0910012107.

Hyysalo, A., Ristola, M., Mäkinen, M.E.L., Häyrynen, S., Nykter, M., and Narkilahti, S. (2017). Laminin α5 substrates promote survival, network formation and functional development of human pluripotent stem cell-derived neurons in vitro. Stem Cell Res. *24*, 118–127. https://doi.org/10.1016/j.scr.2017.09.002.

Kamioka, H., Maeda, E., Jimbo, Y., Robinson, H.P.C., and Kawana, A. (1996). Spontaneous periodic synchronized bursting during formation of mature patterns of connections in cortical cultures. Neurosci. Lett. *206*, 109–112. https://doi.org/10.1016/S0304-3940(96)12448-4.

Kayama, T., Suzuki, I., Odawara, A., Sasaki, T., and Ikegaya, Y. (2018). Temporally coordinated spiking activity of human induced pluripotent stem cell-derived neurons co-cultured with astrocytes. Biochem. Biophysical Res. Commun. *495*, 1028–1033. https://doi.org/10.1016/j.bbrc.2017.11.115.

Kilpinen, H., Goncalves, A., Leha, A., Afzal, V., Alasoo, K., Ashford, S., Bala, S., Bensaddek, D., Casale, F.P., Culley, O.J., et al. (2017). Common genetic variation drives molecular heterogeneity in human iPSCs. Nature *546*, 370–375. https://doi.org/10.1038/nature22403.

Klein Gunnewiek, T.M., van Hugte, E.J.H., Frega, M., Guardia, G.S., Foreman, K.B., Panneman, D., Mossink, B., Linda, K., Keller, J.M., Schubert, D., et al. (2020). Mitochondrial dysfunction impairs human neuronal development and reduces neuronal network activity and synchronicity. Cell Rep., 720227. https://doi.org/10.1101/720227.

Lu, C., Chen, Q., Zhou, T., Bozic, D., Fu, Z., Pan, J.Q., and Feng, G. (2016). Micro-electrode array recordings reveal reductions in both excitation and inhibition in cultured cortical neuron networks lacking Shank3. Mol. Psychiatry *21*, 159–168. https://doi.org/10.1038/mp.2015.173.

Maeda, H., Chiyonobu, T., Yoshida, M., Yamashita, S., Zuiki, M., Kidowaki, S., Isoda, K., Yamakawa, K., Morimoto, M., Nakahata, T., et al. (2016). Establishment of isogenic iPSCs from an individual with SCN1A mutation mosaicism as a model for investigating neurocognitive impairment in Dravet syndrome. J. Hum. Genet. https://doi.org/10.1038/jhg.2016.5.

Mccarthy, K.D. (1980). Preparation of separate astroglial and oligodendroglial cell cultures from rat cerebral tissue. J. Cell Biol. *85*, 890–902.

McConnell, E.R., McClain, M.A., Ross, J., LeFew, W.R., and Shafer, T.J. (2012). Evaluation of multi-well microelectrode arrays for neurotoxicity screening using a chemical training set. NeuroToxicol. *33*, 1048–1057. https://doi.org/10.1016/j.neuro.2012.05.001.

Mertens, J., Marchetto, M.C., Bardy, C., and Gage, F.H. (2016). Evaluating cell reprogramming, differentiation and conversion technologies in neuroscience. Nat. Rev. Neurosci. *17*, 424–437. https://doi.org/10.1038/nrn.2016.46.

Mossink, B., van Rhijn, J.R., Wang, S., Linda, K., Vitale, M.R., Zöller, J.E.M., van Hugte, E.J.H., Bak, J., Verboven, A.H.A., Selten, M., et al. (2021). Cadherin-13 is a critical regulator of GABAergic modulation in human stem-cell-derived neuronal networks. Mol. Psychiatry https://doi.org/10.1038/s41380-021-01117-x.

Napoli, A., and Obeid, I. (2016). Comparative analysis of human and rodent brain primary neuronal culture spontaneous activity using micro-electrode array technology. J. Cell Biochem. *117*, 559–565. https://doi.org/10.1002/jcb.25312.

Novellino, A., Scelfo, B., Palosaari, T., Price, A., Sobanski, T., Shafer, T.J., Johnstone, A.F.M., Gross, G.W., Gramowski, A., Schroeder, O., et al. (2011). Development of micro-electrode array based tests for neurotoxicity: assessment of interlaboratory reproducibility with neuroactive chemicals. Front. Neuroeng. *4*, 1–14. https://doi.org/10.3389/fneng.2011.00004.

Odawara, A., Katoh, H., Matsuda, N., and Suzuki, I. (2016). Physiological maturation and drug responses of human induced pluripotent stem cell-derived cortical neuronal networks in long-term culture. Sci. Rep. *6*, 1–14. https://doi.org/10.1038/srep26181.

Odawara, A., Saitoh, Y., Alhebshi, A.H., Gotoh, M., and Suzuki, I. (2014). Long-term electrophysiological activity and pharmacological response of a human induced pluripotent stem cell-derived neuron and astrocyte co-culture. Biochem. Biophysical Res. Commun. *443*, 1176–1181. https://doi.org/10.1016/j.bbrc.2013.12.142.

Rouhani, F., Kumasaka, N., de Brito, M.C., Bradley, A., Vallier, L., and Gaffney, D. (2014). Genetic background drives transcriptional variation in human induced pluripotent stem cells. PLoS Genet. *10*. https://doi.org/10.1371/journal.pgen.1004432.

Sasaki, T., Suzuki, I., Yokoi, R., Sato, K., and Ikegaya, Y. (2019). Synchronous spike patterns in differently mixed cultures of human iPSC-derived glutamatergic and GABAergic neurons. Biochem. Biophysical Res. Commun. *513*, 300–305. https://doi.org/10.1016/j.bbrc.2019.03.161.

Volpato, V., and Webber, C. (2020). Addressing variability in iPSC-derived models of human disease: guidelines to promote reproducibility. Dis. Models Mech. *13*. https://doi.org/10.1242/dmm.042317.

Wainger, B.J., Kiskinis, E., Mellin, C., Wiskow, O., Han, S.S.W., Sandoe, J., Perez, N.P., Williams, L.A., Lee, S., Boulting, G., et al. (2014). Intrinsic membrane hyperexcitability of amyotrophic lateral sclerosis patient-derived motor neurons. Cell Rep. *7*, 1–11. https://doi.org/10.1016/j.celrep.2014.03.019.

Zhang, Y., Pak, C.H., Han, Y., Ahlenius, H., Zhang, Z., Chanda, S., Marro, S., Patzke, C., Acuna, C., Covy, J., et al. (2013). Rapid single-step induction of functional neurons from human pluripotent stem cells. Neuron *78*, 785–798. https://doi.org/10.1016/j.neuron.2013.05.029.

Stem Cell Reports, Volume *16*

Supplemental Information

Human neuronal networks on micro-electrode arrays are a highly robust tool to study disease-specific genotype-phenotype correlations *in vitro*

Britt Mossink, Anouk H.A. Verboven, Eline J.H. van Hugte, Teun M. Klein Gunnewiek, Giulia Parodi, Katrin Linda, Chantal Schoenmaker, Tjitske Kleefstra, Tamas Kozicz, Hans van Bokhoven, Dirk Schubert, Nael Nadif Kasri, and Monica Frega

Supplemental material

Supplemental items

Figure S1 - Variability in neuronal network phenotypes between control lines.
Figure S2 - Average burst shapes of control lines.
Figure S3 - Connectivity in control neuronal networks.
Figure S4 - Additional parameters affected in MELAS and KS.

Supplemental tables

Table S1 – Explanation of extracted MEA parameters.
Table S2 – Stability of MEA parameters.
Table S3 – Power calculations on MEA data.
Table S4 – Statistics per figure (added as separate excel file)

Supplemental experimental procedures

hiPSC line origin and generation
MEA recordings and data analysis
Principal component analysis

Supplemental figures

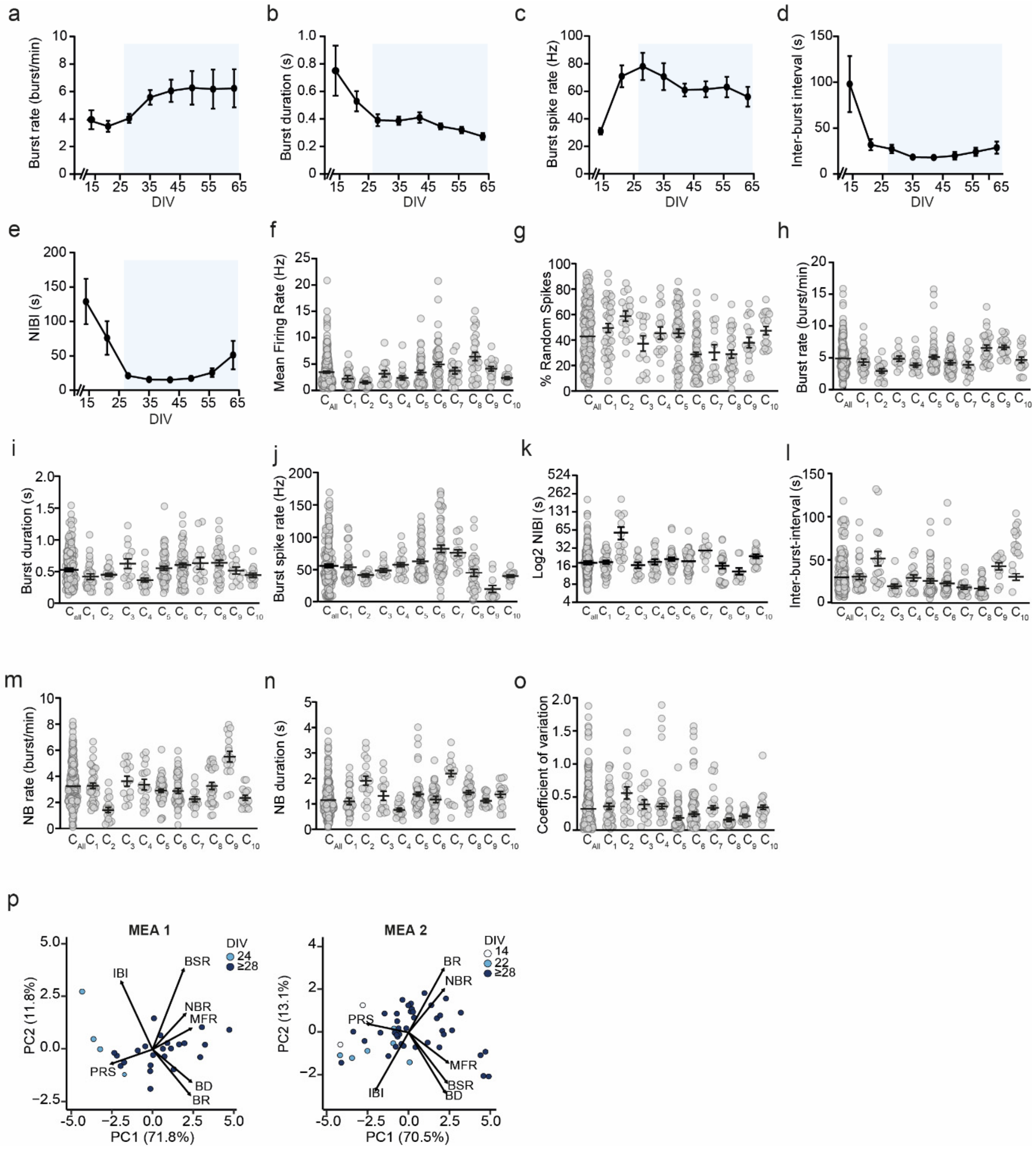

Figure S1. Variability in neuronal network phenotypes between control lines, related to Figure 1.

(**a-e**) Neuronal network parameters (representative line C6) develop to reach a certain plateau after DIV 27 (blue box) for (**a**) BR, (**b**) BD, (**c**) BSR, (**d**) IBI and (**e**) NIBI. (**f-o**) Comparison of the MEA parameters (**f**) MFR, (**g**) PRS, (**h**) BR, (**i**) BD, (**j**) BSR, (**k**) NIBI (on log2 scale), (**l**) IBI, (**m**) NBR, (**n**) NBD, (**o**) CV_{NIBI} between all 10 control lines. Kruskal Wallis Anova with Dunn's correction for multiple testing was used to compare between control lines (**Table S4**). (**p**) PCA plots on all parameters

showing data of one control line (C_6) pooled and color-coded by DIV (two independent MEA plates are analyzed). DIV = days in vitro, BR = mean burst rate, BD = mean burst duration, BSR = burst spike rate, IBI = inter-burst interval, NIBI = Network burst IBI, MFR = mean firing rate, PRS = percentage of random spike, NBR = network burst rate, NBD = network burst duration, CV_{NIBI} = coefficient of variation on NIBI.

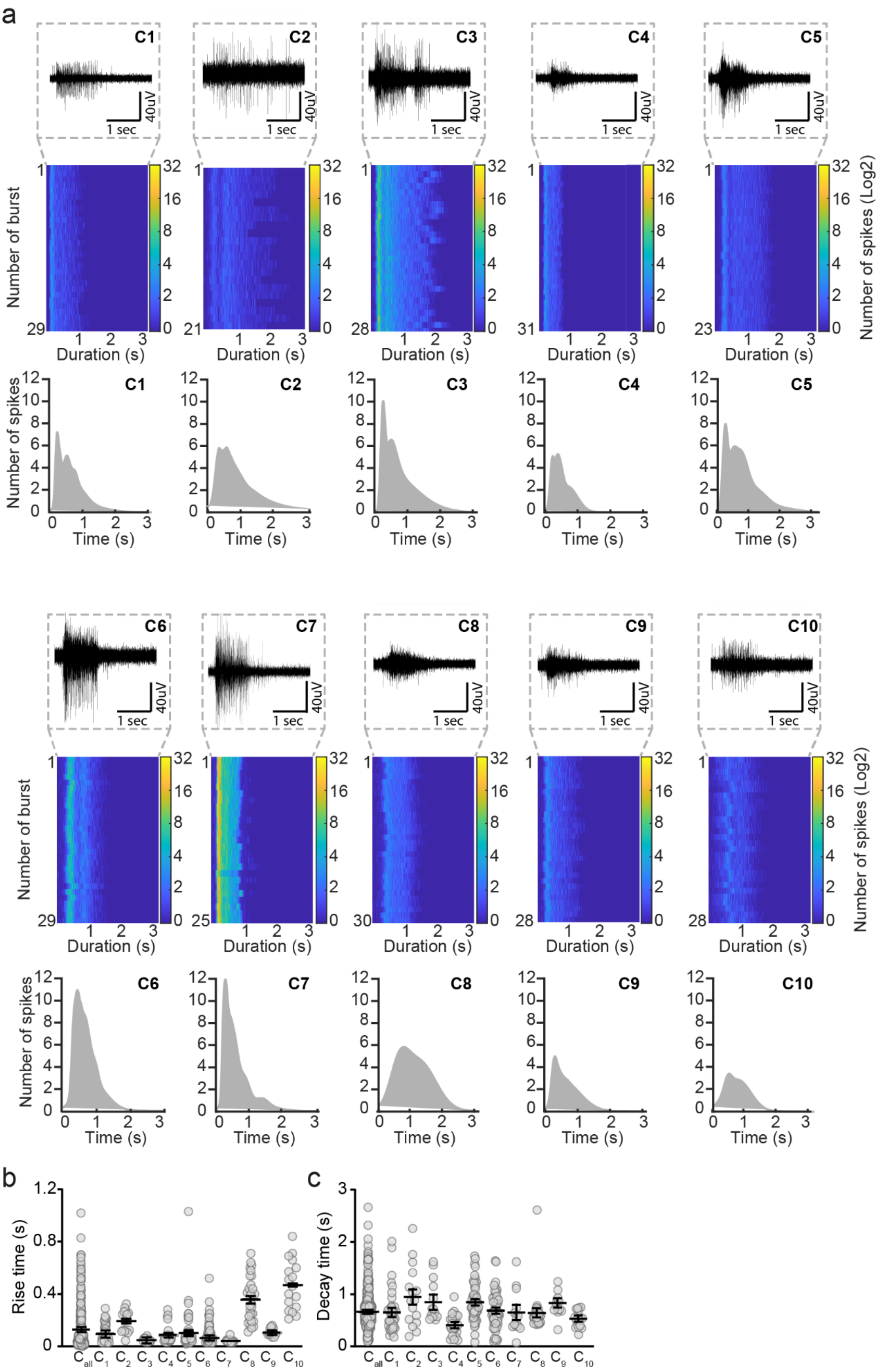
a
C1
C2
C3
C4
C5
C6
C7
C8
C9
C10
1 sec
40uV
Number of burst
Duration (s)
Number of spikes (Log2)
Number of spikes
Time (s)
b
Rise time (s)
c
Decay time (s)

Figure S2. Average burst shapes of control lines, related to Figure 1. (**a**) Representative network burst trace (zoom in) and alignment from one recording of control lines. Bottom panel: average burst shape from all recordings (Sample size n for C_1 = 38, C_2 = 15, C_3 = 14, C_4 = 23, C_5 = 55, C_6 = 58, C_7 = 14, C_8 = 30, C_9 = 12 and C_{10} = 17). Average burst shapes were used to calculate the (**b**) RT and (**c**) DT. Kruskal Wallis ANOVA with Dunn's correction for multiple testing was used to compare between control lines (**Table S4**). RT = Rise time, DT = decay time.

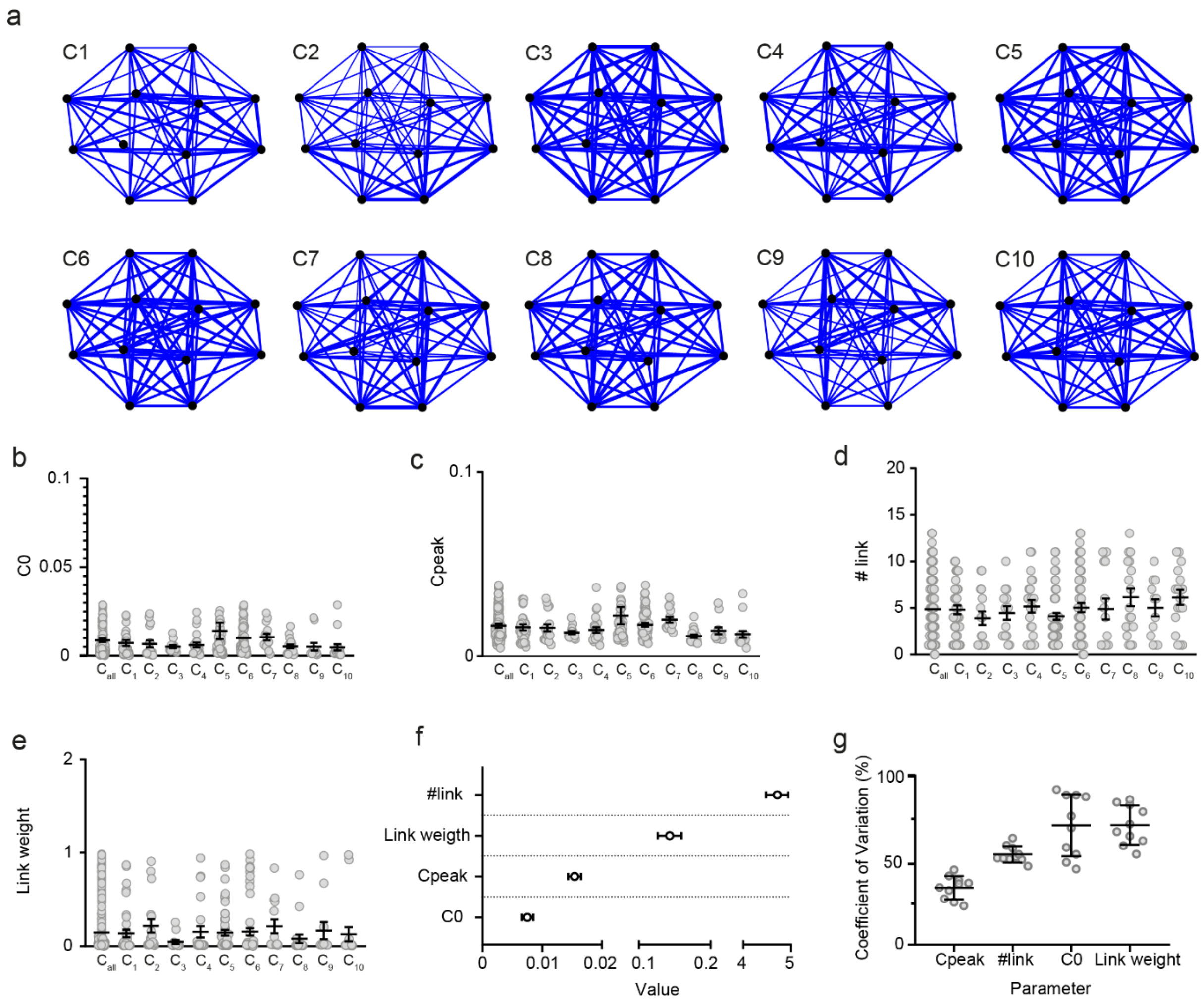

Figure S3. **Connectivity in control neuronal networks, related to Figure 1.** (**a**) Representative connectivity maps showing the links (blue lines) and their strengths (magnitude of the blue lines) (**b-e**) Comparison of the MEA parameters (**b**) C_0, (**c**) C_{peak}, (**d**) number of links and (**e**) weight of links between all 10 control lines. (**f**) Graph showing the range in which MEA parameters C_0, C_{peak}, link weight and number of links of all 10 control lines behave (mean ± 95% confidence interval). Values are first averaged per control line, and then averaged across all control lines. (**g**) Percent coefficient of variation explaining the stability of the respective MEA parameter across all 10 control lines (mean ± standard deviation of the mean). N = 278 wells. Kruskal Wallis ANOVA with Dunn's correction for multiple testing was used to compare between control lines (**Table S4**).

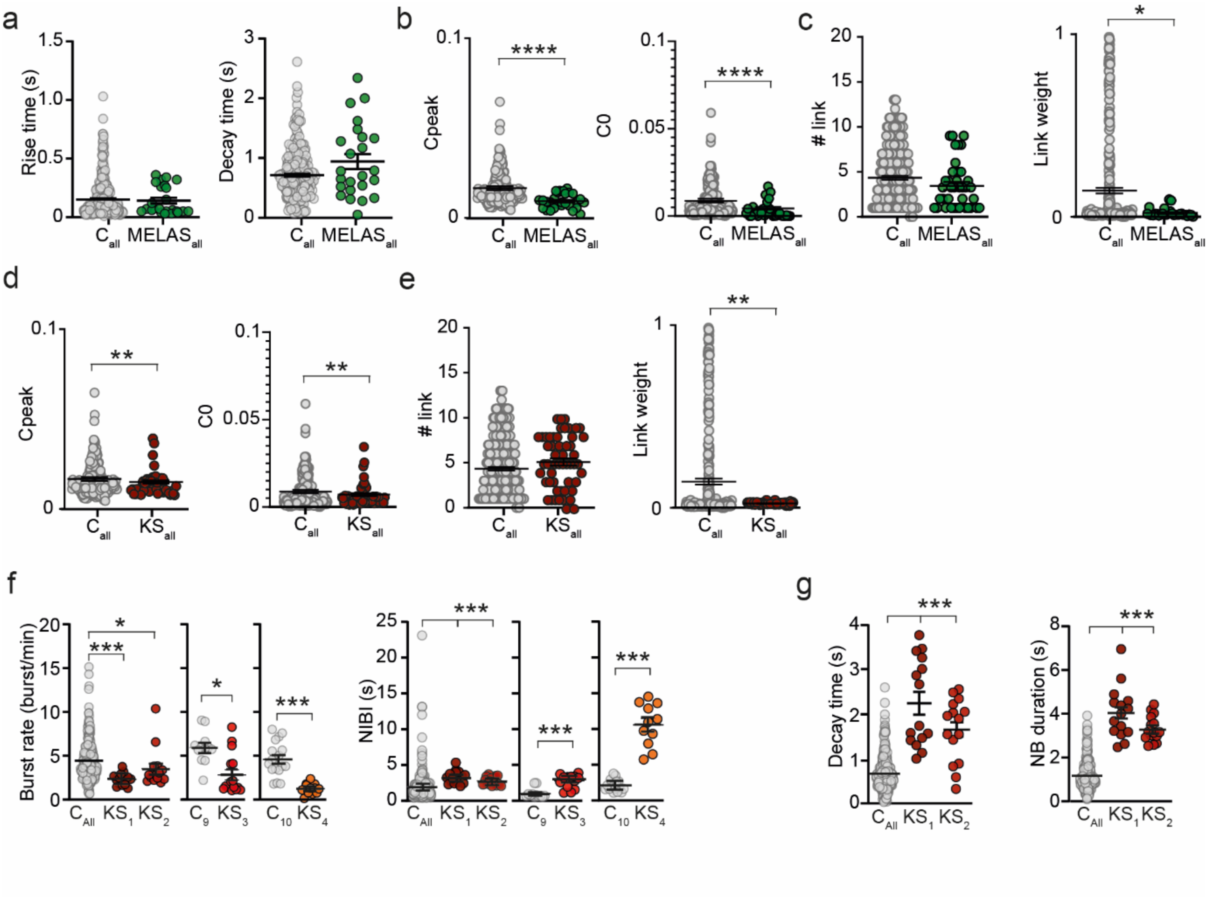

Figure S4. **Additional parameters affected in MELAS and KS, related to Figure 3.** (**a-c**) Comparison of MEA parameters (**a**) RT and DT, (**b**) C_{peak}, C_0 and (**c**) link number and link weight for MELAS samples versus all controls (Mann Whitney U test with Bonferroni correction for multiple testing was used to compare between patient lines and controls). (**d-e**) Comparison of MEA parameters (**d**) C_{peak}, C_0 and (**e**) link number and link weight for KS samples versus all controls (Mann Whitney U test with Bonferroni correction for multiple testing was used to compare between patient lines and controls). (**f-g**) Comparison of MEA parameters (**f**) BR and NIBI for all KS_1 and KS_2 compared to all controls, and for KS_3 and KS_4 compared to their isogenic controls and (**g**) DT and NBD for KS_1 and KS_2 compared to all controls (Kruskal Wallis Anova with Dunn's correction for multiple testing was used to compare between control lines). (**Table S4**). *p* = 0.05 *, *p* = 0.01 **, *p* = 0.001 *** and *p*<0.0001 ****. DIV = days in vitro, BR = mean burst rate, NIBI = Network burst inter-burst-interval, RT = Rise time, DT = decay time, BD = burst duration, NBR = network burst rate, NBD = network burst duration. All means, *p*-values and used statistic tests are reported in Table S4.

Supplemental tables

Parameter	Short name	Unit	Explanation
Mean firing rate	MFR	Spikes/s	The MFR is a measure of all spikes detected in each electrode during time, which is averaged for all 12 electrodes in a well.
Percentage of random spikes	PRS	%	The PRS represents the percentage of all spikes that are not organized into a random burst, or a network burst.
Mean burst rate	MBR	Burst/ minute	A burst is detected when at least 4 spikes are 50 ms or less spaced from each other. The MBR is a measure of all burst detected in each channel in time, which is averaged for all 12 electrodes in a well.
Mean burst duration	MBD	s	The MBD represents the duration of the burst.
Inter-burst interval	IBI	s	The IBI the interval between two consecutive bursts.
Burst spike rate	BSR	Spikes/s	The BSR is the number of spikes detected in a burst divided by the duration of the burst.
Network burst rate	NBR	Network burst/ minute	When a burst is occurring in more than 6 channels at the same time, and 6 are time locked, these bursts are classified as network burst.
Network burst duration	NBD	s	The NBD represents the duration of the network burst.
Network inter-burst interval	NIBI	s	The NIBI is the interval between two consecutive network bursts.
Coefficient of variation on NIBI	CV_{NIBI}		The coefficient of variation is calculated by dividing the standard deviation of all NIBIs to the mean. The value ranges between 0 (very regular network burst) to 1 (very irregular network burst).
Average burst shape			The average burst shape is an averaged histogram representing the number of spikes occurring into all network burst detected in one well.
Rise time	RT	s	The rise time is extracted by the average burst shape histogram by fitting it with a Gaussian and calculating the slope between the 20 and 80% of the peak on the rising edge.
Decay time	DT	s	The decay time is extracted by the average burst shape histogram by fitting it with a Gaussian and calculating the slope between 80 and 20% of the peak on the falling edge.
C_0			C_0 represent the value of the cross correlogram function in the central time bin and indicates the degree of synchronization between two electrodes.
C_{peak}			C_{peak} represent the peak value of the cross correlogram and indicates the degree of correlation between two electrodes.
Number of links			The number of links indicates the total number of connections between all MEAs electrodes.
Link weight			The link weight indicates the averaged strength of all links detected between all MEAs electrodes

Supplemental table 1: **Explanation of extracted MEA parameters.**

Panel		*MFR*	*PRS*	*MBR*	*MBD*	*BSR*	*IBI*	*NBR*	*NBD*	*NIBI*	*CV_{NIBI}*	*RT*	*DT*
i	Mean	3.5	41.3	4.8	0.51	58.5	27.47	3.2	1.28	22.68	0.3	0.15	0.71
	SEM	0.2	1.3	0.1	0.02	1.8	1.20	0.1	0.04	1.16	0.0	0.01	0.02
	Min	0.4	3.0	1.0	0.12	8.5	5.08	0.3	0.19	7.85	0.0	0.01	0.07
	Max	20.9	92.3	15.5	1.53	170.2	131.99	7.0	4.02	222.80	1.9	1.03	2.61
j	Mean	62.96	46.37	39.38	42.88	39.63	58.21	38.37	40.60	43.79	74.77	58.49	48.57
	STD	17.39	14.53	9.337	7.749	20.14	13.34	9.218	10.56	9.092	18.73	26.93	12.46
	Min	29.38	26.40	26.37	28.63	17.73	33.71	28.25	20.10	30.54	47.21	28.61	29.27
	Max	87.68	73.74	57.78	51.33	84.03	80.75	61.30	54.24	93.80	103.7	141.3	64.41

		C_{peak}	*C_0*	*# link*	*Link weight*
f	Mean	0.007	0.004	4.712	0.143
	SEM	0.001	0.0009	0.234	0.016
	Min	0.010	0.004	3.647	0.045
	Max	0.022	0.014	5.889	0.216
g	Mean	46.79	75.3	60.86	76.1
	STD	13.75	26.57	9.736	13.13
	Min	25.45	48.76	56.98	63.7
	Max	67.95	92.5	75.05	85.1

Supplemental table 2: Stability of MEA parameters, related to Figure 1i-j, 3f-g: Values of MEA parameters presented **in Figure 1i, j** and **Figure S3f, g. Panel i, f:** Range of each MEA parameters in which controls behave**. Panel j, g:** Percentage of variation of each MEA parameter**.**

		Post hoc power calculation		*A priori power calculation*	
Compared lines	**Top parameters**	**# wells used for calculation**	**Power**	**# wells needed for power 0.9**	**Effect size**
C9 vs KS3	BR	C9 = 12	0.999	12	1.9
	BD	KS3 =16	0.999	12	1.9
	NBR		1	6	3
	NBD		0.999	8	2.7
	NIBI		0.999	10	2.3
C10 vs KS4	MFR	C10 = 17	0.999	12	2
	BR	KS4 =12	1	6	3
	NBR		0.98	14	2
	NBD		0.97	8	2.8
	NIBI		1	6	3.4
C4 versus M2	MFR	C4 = 23	0.999	12	2
	PRS	M2 = 23	1	10	2.1
	BD		0.998	20	1.4
	BSR		0.999	16	1.5
	NBR		1	6	3
C5 versus M3	MFR	C5 = 55	1	12	1.9
	PRS	M3 = 68	1	12	1.9
	BD		0.999	24	1.4
	BRS		1	22	1.3
	NBR		1	12	2
C2 versus M1	PRS	C2 = 17	1	8	2.8
	BD	M1 = 24	0.999	16	1.6
	NBR		0.927	6	1
	BSR		0.997	22	1.5
All controls vs KS	BR	C_{all} = 278	1	14	2
	BD	KS_{all} = 58	1	12	2.1
	NBR		1	14	2
	NBD		1	14	1.9
	NIBI		0.999	10	2.55
	DT		0.999	12	2.27
All controls vs MELAS	MFR	C_{all} = 278	1	12	1.9
	PRS	$MELAS_{all}$ = 115	1	8	2.5
	BD		1	8	2.78
	BSR		1	10	2.3
	NBR		1	6	3

Supplemental table 3: Power calculations on MEA data. Left: post hoc power calculation on PCA parameters that describe the patient phenotype. Alpha is 0.05. Right: a priori Power calculation to calculate the sample size needed to achieve a power of 0.9 using the PCA parameters that describe the patient phenotype. Effect size represents the magnitude of the difference between populations.

Supplemental table 4 (added as separate excel file): Statistics per figure. Sample size n represents the number of recorded MEA wells between DIV27-35. All data represent means ± SEM. Statistical tests used per figure are reported in the figure legends.

Supplemental experimental procedures

hiPSC line origin and generation

C_1 originated from a 36-year old female (Kondo et al., 2017; Okita et al., 2011), reprogrammed using episomal vector-based reprogramming of the Yamanaka transcription factors *Oct4*, *c-Myc*, *Sox2* and *Klf4* (Takahashi & Yamanaka, 2006), showing no karyotypical malformations. Control line **C_6** was derived from a 30-year old male (Mandegar et al., 2016; Miyaoka et al., 2014) and reprogrammed using episomal vector-based reprogramming of the Yamanaka factors and was tested for genetic integrity using SNP assay (Frega et al., 2019). Control line **C_7** was derived from a 41-year-old-female and reprogrammed using the Simplicon^{TM} reprogramming kit (Millipore). Overexpression of the Yamanaka factors was introduced by a non-integrative, non-viral one-step transfection. Genomic stability was checked using STR analysis. Control line **C_8** was derived from a 9-year old male and reprogrammed using retroviral-vector based reprogramming of the Yamanaka factors and tested for pluripotency and genomic integrity based on SNP arrays.

The KS isogenic patient-control hiPSC set consisting of control line **C_9 and KS_3** was derived from a 34-year old female with a mosaic heterozygous 233 kbp deletion of chromosome 9, including the *EHMT1* and *CACNA1B* gene, diagnosed with KS (Frega et al., 2019). Both clones were reprogrammed using retro-viral vectors expressing the Yamanaka factors and tested for genomic integrity based on SNP array (Frega et al., 2019). The second KS isogenic patient-control hiPSC set, consisting of control line **C_{10} and KS_4** was previously derived from a healthy 51-year old male and reprogrammed using expression of Yamanaka factors by non-integrating Sendai virus. KS_4 was generated using CRISPR/Cas 9 technology to induce a heterozygous *EHMT1* mutation to mimic KS, as described previously, producing an isogenic set. Both lines were tested for genomic integrity based on SNP array (Frega et al., 2019). In addition, we included two KS patient hiPSC lines, **KS_1 and KS_2,** which were previously characterized and derived from a 13-year old and a 12-year-old female, respectively, diagnosed with KS. HiPSC clones were obtained by reprogramming of retroviral expression of the Yamanaka factors (Frega et al., 2019).

The MELAS syndrome isogenic patient-control hiPSC sets consisting of control line **C_2, C_3, C_4 and C_5 and patient lines M_1 and M_2** were generous gifts form Esther-Perales Clemente and Timothy Nelson. **C_2 and M_1** were derived from a 17-year-old female, and **C_4 and M_2** form a 45-year-old female. Both donors were diagnosed with MELAS syndrome, harboring the pathogenic variant m.3243A>G. Reprogramming of fibroblasts to hiPSCs resulted in clones with varying levels of m.3243A>G heteroplasmy (Klein Gunnewiek et al., 2020; Perales-Clemente et al., 2016). Control lines C_2 and C_4 originated from hiPSC clones with confirmed 0% heteroplasmy, while M_1 and M_2 had 66% of m.3243A>G heteroplasmy and 80% heteroplasmy, respectively. Control line **C_3** was derived from a 30-

year-old male with MELAS syndrome and was confirmed to have 0% heteroplasmy upon reprogramming to hiPSCs. All lines were reprogrammed using CytoTune-iPS Sendai Reprogramming Kits according to manufacturer's instructions (Invitrogen, A13780-02, A16517, A16518) and were previously characterized and had a normal karyotype (Perales-Clemente et al., 2016). MELAS syndrome isogenic patient-control hiPSC set, consisting of control line **C_5 and M_3** was derived from a 42-year-old male with MELAS syndrome with a pathogenic variant m.3243A>G. Reprogramming through lentiviral transduction of the Yamanaka transcription factors resulted in hiPSC clones with varying levels of m.3243A>G heteroplasmy. C_5 originated from an hiPSC clone with confirmed 0% of m.3243A>G heteroplasmy and M_3 originated from an hiPSC clone with 70% heteroplasmy. Both C_5 and M_3 were previously characterized and had normal karyotypes (Klein Gunnewiek et al., 2020).

MEA recordings and data analysis

Data analysis using Multiwell-Analyzer. The mean firing rate (MFR) (Hz) was calculated for each well individually by averaging the firing rate of each separate channel by all the active channels of the well. Bursts were detected using the Multiwell analyzer build-in burst detection algorithm. The algorithm was set to define bursts if 4 spikes were in close proximity with a maximum of 50 ms inter spike interval (ISI) to start a burst, and a maximum of 50 ms ISI to end a burst, with a minimum of 100 ms inter burst interval (IBI). Network bursts were defined when at least 50% of all channels simultaneously displayed a burst. In rare cases, we observed synchronous events which were not properly identified as network bursts **(Fig. 2g)**. It was however clear that the observed pattern of the synchronous events was similar to control networks in which network bursts were properly detected. When the network burst detection was insufficient the network bursts detection was decreased down to, but not further than at least 25% of all channels participating in the network burst. The percentage of random spikes (PRS) was defined by calculating the percentage of spikes that neither belonged to a burst nor a network burst. The IBI, and network burst IBI (NIBI) were calculated by the subtraction of the time stamp of the beginning of each burst or network burst, respectively, from the time stamp of the ending of the previous. To calculate the IBI, the mean IBI per channel was calculated and averaged across all channels. The NIBI is calculated by averaging the found NIBIs in one well. To explain the regularity at which a network burst occurs, the coefficient of variation (CV) was calculated by calculating the standard deviation of all NIBIs and dividing it by the mean of all NIBIs.

Analysis of the average burst shape. The generation of average burst shapes for each control was performed using adapting scripts and functions implemented in MATLAB (Melé and Rinn, 2016; Van De Vijver et al., 2019). Spike trains containing all events detected in the 12 electrodes were binned. Network burst were detected from a spike train containing the events present in all channels

by using an inter-spike interval threshold (30 ms). All network bursts that have a duration of less than 100 ms were removed from the detection. For each well, the average burst shape was calculated by aligning all the network bursts detected to the longest network burst and by summing all spikes (bin: 10 ms). Then, we calculated the average burst shape for each line by averaging all the histograms of the individual wells. We fitted the curves with Gaussian distribution to obtain smoother profiles using the build in fit function. The rise and decay times were calculated as the absolute difference between the time at 20% and 80% before and after the peak, respectively. Slopes of rise and decay time were subsequently calculated using the build in polyfit and polyval functions.

Correlation analysis. We obtained connectivity matrix for each culture by applying the Filtered Cross-Correlation algorithm (Pastore, Massobrio, et al., 2018) available in the free software SPICODYN (Pastore, Godjoski, et al., 2018). Cross correlograms were obtained using a bin size of 0.1 ms and a correlation window of 150 ms. Matrices were then analyzed using custom script in MATLAB. We evaluated the C_{peak} (i.e. maximum value of the -cross-correlation function), C_0 (i.e. value of the cross-correlation function in the central bin), total number of links (i.e. the number of functional connections within the nodes of the same network) and the link weight (i.e. the average strength of the identified connections).

Principal component analysis

Principal component analysis (PCA) was performed on data from 12 MEA parameters (MFR, PRS, BR, BD, BSR, IBI, NBR, NBD, NIBI, CV_{NIBI}, RT, and DT, see Table S1) for all control samples ($n_{wells} = 278$, $N_{plates} = 47$). Separately, PCA was performed for controls including samples from either KS ($n_{wells} = 58$, $N_{plates} = 9$) or MELAS ($n_{wells} = 112$, $N_{plates} = 23$) neurons. PCA on KS versus control samples was performed on data from all 12 MEA parameters. PCA was performed for each KS isogenic patient-control set separately as well, using the same MEA parameters. PCA on MELAS versus control samples was performed on data from 7 MEA parameters (MFR, PRS, BR, BD, BSR, IBI, and NBR), since many samples did not show network bursts (i.e. NBD, NIBI, CV_{NIBI}, RT, and DT could not be calculated). PCA was performed for each MELAS isogenic patient-control set separately as well, using the same MEA parameters.

PCA in **Fig. 2i** was performed for samples from line C1 separately to test the effect of astrocyte batch and MEA plate, using data from all 12 MEA parameters. Samples from C1 were measured using three different astrocyte batches and 2-3 different MEA plates per astrocyte batch. PCA was performed on all C1 samples ($n_{wells} = 27$, $N_{plates} = 7$, $N_{astro} = 3$) to check the effect of astrocyte batch and MEA batch. ANOVA was used to evaluate the significance of the linear models (parameter ~ MEA plate and parameter ~ astrocyte batch) to determine the effect of astrocyte batch and MEA batch,

separately, on each MEA parameter. Statistics are reported in **Table S4**. In **Fig. 2j** the percentage of variance explained by astrocyte batch and MEA batch on each parameter is shown for all parameters, calculated by dividing the sum of squares by the total sum of squares (x 100) for each linear model separately.

Furthermore, PCA in **Fig. S1p** was performed for samples from line C6 that were measured up to DIV62, to check whether samples from early DIVs cluster away from samples at a higher DIVs. This was performed for two MEA plates separately, for which data was available between DIV14-56 (n_{wells} = 7) and DIV24-62 (n_{wells} = 4), using data from 7 MEA parameters (MFR, PRS, BR, BD, BSR, IBI, and NBR), since at an early time point (almost) no network bursts are detected (i.e. NBD, NIBI, CV_{NIBI}, RT, and DT could not be calculated).

References

Frega, M., Linda, K., Keller, J. M., Gümüş-Akay, G., Mossink, B., van Rhijn, J. R., Negwer, M., Klein Gunnewiek, T., Foreman, K., Kompier, N., Schoenmaker, C., van den Akker, W., van der Werf, I., Oudakker, A., Zhou, H., Kleefstra, T., Schubert, D., van Bokhoven, H., & Nadif Kasri, N. (2019). Neuronal network dysfunction in a model for Kleefstra syndrome mediated by enhanced NMDAR signaling. *Nature Communications*, *10*(1), 1–15. https://doi.org/10.1038/s41467-019-12947-3

Klein Gunnewiek, T. M., Hugte, E. J. H. Van, Frega, M., Guardia, G. S., Foreman, K. B., Panneman, D., Mossink, B., Linda, K., Keller, J. M., Schubert, D., Cassiman, D., Morava, E., Rodenburg, R., Perales-Clemente, E., Nelson, T. J., Kasri, N. N., & Kozicz, T. (2020). Mitochondrial dysfunction impairs human neuronal development and reduces neuronal network activity and synchronicity. *Cell Reports*, 720227. https://doi.org/10.1101/720227

Kondo, T., Imamura, K., Funayama, M., Tsukita, K., Miyake, M., Ohta, A., Woltjen, K., Nakagawa, M., Asada, T., Arai, T., Kawakatsu, S., Izumi, Y., Kaji, R., Iwata, N., & Inoue, H. (2017). iPSC-Based Compound Screening and In Vitro Trials Identify a Synergistic Anti-amyloid β Combination for Alzheimer's Disease. *Cell Reports*, *21*(8), 2304–2312. https://doi.org/10.1016/j.celrep.2017.10.109

Mandegar, M. A., Huebsch, N., Frolov, E. B., Shin, E., Truong, A., Olvera, M. P., Chan, A. H., Miyaoka, Y., Holmes, K., Spencer, C. I., Judge, L. M., Gordon, D. E., Eskildsen, T. V., Villalta, J. E., Horlbeck, M. A., Gilbert, L. A., Krogan, N. J., Sheikh, S. P., Weissman, J. S., … Conklin, B. R. (2016). CRISPR Interference Efficiently Induces Specific and Reversible Gene Silencing in Human iPSCs. *Cell Stem Cell*, *18*(4), 541–553. https://doi.org/10.1016/j.stem.2016.01.022

Miyaoka, Y., Chan, A. H., Judge, L. M., Yoo, J., Huang, M., Nguyen, T. D., Lizarraga, P. P., So, P. L., & Conklin, B. R. (2014). Isolation of single-base genome-edited human iPS cells without antibiotic selection. *Nature Methods*, *11*(3), 291–293. https://doi.org/10.1038/nmeth.2840

Okita, K., Matsumura, Y., Sato, Y., Okada, A., Morizane, A., Okamoto, S., Hong, H., Nakagawa, M.,

Tanabe, K., Tezuka, K. I., Shibata, T., Kunisada, T., Takahashi, M., Takahashi, J., Saji, H., & Yamanaka, S. (2011). A more efficient method to generate integration-free human iPS cells. *Nature Methods*, *8*(5), 409–412. https://doi.org/10.1038/nmeth.1591

Pastore, V. P., Godjoski, A., Martinoia, S., & Massobrio, P. (2018). SpiCoDyn: A Toolbox for the Analysis of Neuronal Network Dynamics and Connectivity from Multi-Site Spike Signal Recordings. *Neuroinformatics*, *16*(1), 15–30. https://doi.org/10.1007/s12021-017-9343-z

Pastore, V. P., Massobrio, P., Godjoski, A., & Martinoia, S. (2018). *Identification of excitatory-inhibitory links and network topology in large-scale neuronal assemblies from multi-electrode recordings*. https://doi.org/10.1371/journal.pcbi.1006381

Perales-Clemente, E., Cook, A. N., Evans, J. M., Roellinger, S., Secreto, F., Emmanuele, V., Oglesbee, D., Mootha, V. K., Hirano, M., Schon, E. A., Terzic, A., & Nelson, T. J. (2016). Natural underlying mt DNA heteroplasmy as a potential source of intra-person hi PSC variability . *The EMBO Journal*, *35*(18), 1979–1990. https://doi.org/10.15252/embj.201694892

Takahashi, K., & Yamanaka, S. (2006). Induction of Pluripotent Stem Cells from Mouse Embryonic and Adult Fibroblast Cultures by Defined Factors. *Cell*, *126*(4), 663–676. https://doi.org/10.1016/j.cell.2006.07.024
